# MaxGIRF: Image reconstruction incorporating concomitant field and gradient impulse response function effects

**DOI:** 10.1002/mrm.29232

**Published:** 2022-04-21

**Authors:** Nam G. Lee, Rajiv Ramasawmy, Yongwan Lim, Adrienne E. Campbell‐Washburn, Krishna S. Nayak

**Affiliations:** ^1^ Department of Biomedical Engineering University of Southern California Los Angeles California USA; ^2^ Cardiovascular Branch, Division of Intramural Research, National Heart, Lung, and Blood Institute National Institutes of Health Bethesda Maryland USA; ^3^ Ming Hsieh Department of Electrical and Computer Engineering University of Southern California Los Angeles California USA

**Keywords:** concomitant fields, expanded signal model, gradient distortion, gradient impulse response function, MRI reconstruction

## Abstract

**Purpose:**

To develop and evaluate an improved strategy for compensating concomitant field effects in non‐Cartesian MRI at the time of image reconstruction.

**Theory:**

We present a higher‐order reconstruction method, denoted as MaxGIRF, for non‐Cartesian imaging that simultaneously corrects off‐resonance, concomitant fields, and trajectory errors without requiring specialized hardware. Gradient impulse response functions are used to predict actual gradient waveforms, which are in turn used to estimate the spatiotemporally varying concomitant fields based on analytic expressions. The result, in combination with a reference field map, is an encoding matrix that incorporates a correction for all three effects.

**Methods:**

The MaxGIRF reconstruction is applied to noiseless phantom simulations, spiral gradient‐echo imaging of an International Society for Magnetic Resonance in Medicine/National Institute of Standards and Technology phantom, and axial and sagittal multislice spiral spin‐echo imaging of a healthy volunteer at 0.55 T. The MaxGIRF reconstruction was compared against previously established concomitant field‐compensation and image‐correction methods. Reconstructed images are evaluated qualitatively and quantitatively using normalized RMS error. Finally, a low‐rank approximation of MaxGIRF is used to reduce computational burden. The accuracy of the low‐rank approximation is studied as a function of minimum rank.

**Results:**

The MaxGIRF reconstruction successfully mitigated blurring artifacts both in phantoms and in vivo and was effective in regions where concomitant fields counteract static off‐resonance, superior to the comparator method. A minimum rank of 8 and 30 for axial and sagittal scans, respectively, gave less than 2% error compared with the full‐rank reconstruction.

**Conclusions:**

The MaxGIRF reconstruction simultaneously corrects off‐resonance, trajectory errors, and concomitant field effects. The impact of this method is greatest when imaging with longer readouts and/or at lower field strength.

## Abstract 2

Click here for author‐reader discussions

## 1 INTRODUCTION

Image quality from MRI that uses non‐Cartesian sampling, particularly spirals, has improved continuously over the past 30 years. Current state‐of‐the‐art spiral MRI provides quality that is comparable to its 2D/3D Cartesian counterparts, and is appropriate for clinical use.^1^, ^2^ Spiral acquisitions are attractive because they provide high scan and SNR efficiency, robustness to motion artifacts, and are advantageous for fast imaging applications such as MR fingerprinting^3^, ^4^ and cardiac imaging.^5^

Spiral imaging requires overcoming unique challenges, notably off‐resonance, gradient distortion, and concomitant field effects. The first two effects are well known in the literature; static off‐resonance leads to local blurring, and gradient distortion results in trajectory errors that manifest themselves as halo artifacts near edges. The effects of concomitant fields are less widely recognized, but are extremely important for long readouts, scan planes farther from isocenter, and at low B_0_ field strengths. Concomitant fields constitute an additional nonrotating magnetic field ($B_{x}$, $B_{y}$) in the laboratory reference frame whenever linear gradients are active.^6^ Spatial encoding in MRI is achieved by the Larmor frequency, which is proportional to the magnitude of the applied magnetic field. The applied magnetic field is a superposition of the homogeneous ($B_{o}$) main magnetic field and the transverse $\left(B_{x}\left(t\right),,,B_{y}\left(t\right)\right)$ field and longitudinal field $\left({\mathrm{dB}}_{z}(t)\right)$ produced by a gradient coil. The dot product of three gradient fields $G\left(t\right)={\left(\frac{dB_{z}\left(t\right)}{\mathrm{dx}},,,\frac{dB_{z}\left(t\right)}{\mathrm{dy}},,,\frac{dB_{z}\left(t\right)}{\mathrm{dz}}\right)}^{T}$ with a spatial position causes a linear frequency offset. In contrast, the transverse component contributes a nonlinear, higher‐order frequency offset, which is represented as a sum of products of quadratic gradients with higher‐order spatial terms (eg, $G_{y,i}\left(t\right)G_{z,i}\left(t\right)\mathrm{xz}$). Therefore, spiral imaging accrues a spatiotemporally varying phase due to concomitant fields in addition to static off‐resonance.^7^

Several previous works successfully mitigated concomitant field effects by means of image reconstruction method.^7^, ^8^, ^9^ King et al^7^ proposed a concomitant field‐correction method based on frequency‐segmented deblurring, referred to here as King's method. This approach uses approximations to separate the concomitant field phase into a time‐dependent parameter consisting of the time integral of common gradient terms and the rest as a time‐independent frequency offset. King's method then performs frequency‐segmented deblurring. Two recent approaches by Chen et al^8^ and Cheng et al^9^ achieved a more computationally efficient reconstruction and simultaneously corrected static off‐resonance and concomitant fields based on King's approximations.

Wilm et al^10^ proposed a powerful general approach using NMR field probes^11^, ^12^ in conjunction with a higher‐order encoding model, which inspires this work. This approach incorporates higher‐order dynamic fields to the encoding process and has demonstrated excellent image quality for several applications, including diffusion^10^, ^13^ and structural imaging.^14^ A dynamic field camera^15^, ^16^ consisting of spatially distributed NMR field probes is used to measure phase evolutions at various positions for high‐order field expansions with globally smooth functions.^10^, ^13^, ^17^ The NMR field probes provide real‐time monitoring of field evolutions from various sources; however, commercially available systems are fairly expensive, and building an in‐house system from scratch requires expertise beyond most MRI labs.^11^, ^16^, ^18^, ^19^, ^20^ Therefore, although very promising, the higher‐order approach relying on field‐camera measurements is not widely available.

The characterization of gradient distortions with gradient impulse response functions (GIRFs)^21^, ^22^ can be a reasonable surrogate for NMR field probes. Assuming a linear time invariant system model for the gradient chain, GIRFs capture gradient delays, eddy current effects, and mechanically induced field oscillations. For each gradient axis, an MR system is perturbed with a set of input gradients, and field responses are measured with either a dynamic field camera or phantom‐based methods. Field‐camera measurements provide both self‐responses and cross‐responses (eg, input gradient on the *x*‐axis and field response on the *y*‐axis) in a single measurement, thereby allowing the full characterization of a multiple‐input, multiple‐output linear time invariant system.^22^, ^23^ On the other hand, phantom‐based methods typically measure only self‐term GIRFs^24^ and $B_{0}$ cross‐terms.^25^ Phantom‐based GIRFs have proven to be effective in several applications, including RF pulse design,^26^, ^27^ eddy current–induced artifact correction,^28^ and image reconstruction.^29^, ^30^, ^31^ Therefore, the phantom‐based method appears to be a reasonable compromise to an accurate, albeit expensive field monitoring device.

In this work, because concomitant fields are analytically expressed with gradients and spatial coordinates, we hypothesize that gradients predicted with phantom‐based GIRFs can better estimate concomitant fields than nominal gradients. Following this, we propose a novel higher‐order image reconstruction method, denoted as MaxGIRF, which incorporates concomitant fields, static off‐resonance, and GIRF trajectory corrections. The “Max” part of the MaxGIRF acronym reflects the fact that the concomitant fields are also known as “Maxwell fields” in the literature, because they are based on the principles of electromagnetism described by Maxwell's equations. This proposed framework can be considered as a surrogate to NMR field probes that require no special hardware but require a good analytic model of concomitant fields that depends on coil geometry^6^, ^32^ and severity of gradient nonlinearity.^33^ Non‐Cartesian imaging with long readouts generally benefits from this method, but its impact will be greatest at high‐performance low‐field systems,^34^, ^35^ because the effect of concomitant fields scales quadratically with the maximum gradient amplitude and inversely to the main magnetic field $B_{0}$.

We first validate the proposed method using noiseless simulations at various field strengths and off‐center positions. A guideline for selecting an optimal rank is provided when a low‐rank approximation is applied to the MaxGIRF encoding model. The MaxGIRF reconstructions using nominal and GIRF‐predicted gradients are compared at 0.55 T using an International Society for Magnetic Resonance in Medicine (ISMRM)/National Institute of Standards and Technology (NIST) system phantom. Finally, MaxGIRF reconstructions are demonstrated in vivo using axial and sagittal spiral spin‐echo data of the head and neck, and made available open source.

## 2 THEORY

In this work, we address sequences in which the net phase of all isochromats within a voxel before the next RF pulse can be ignored, such as any pulse sequences with spoiler gradients at the end of each TR.

### 2.1 The MaxGIRF encoding

**FIGURE 1:**
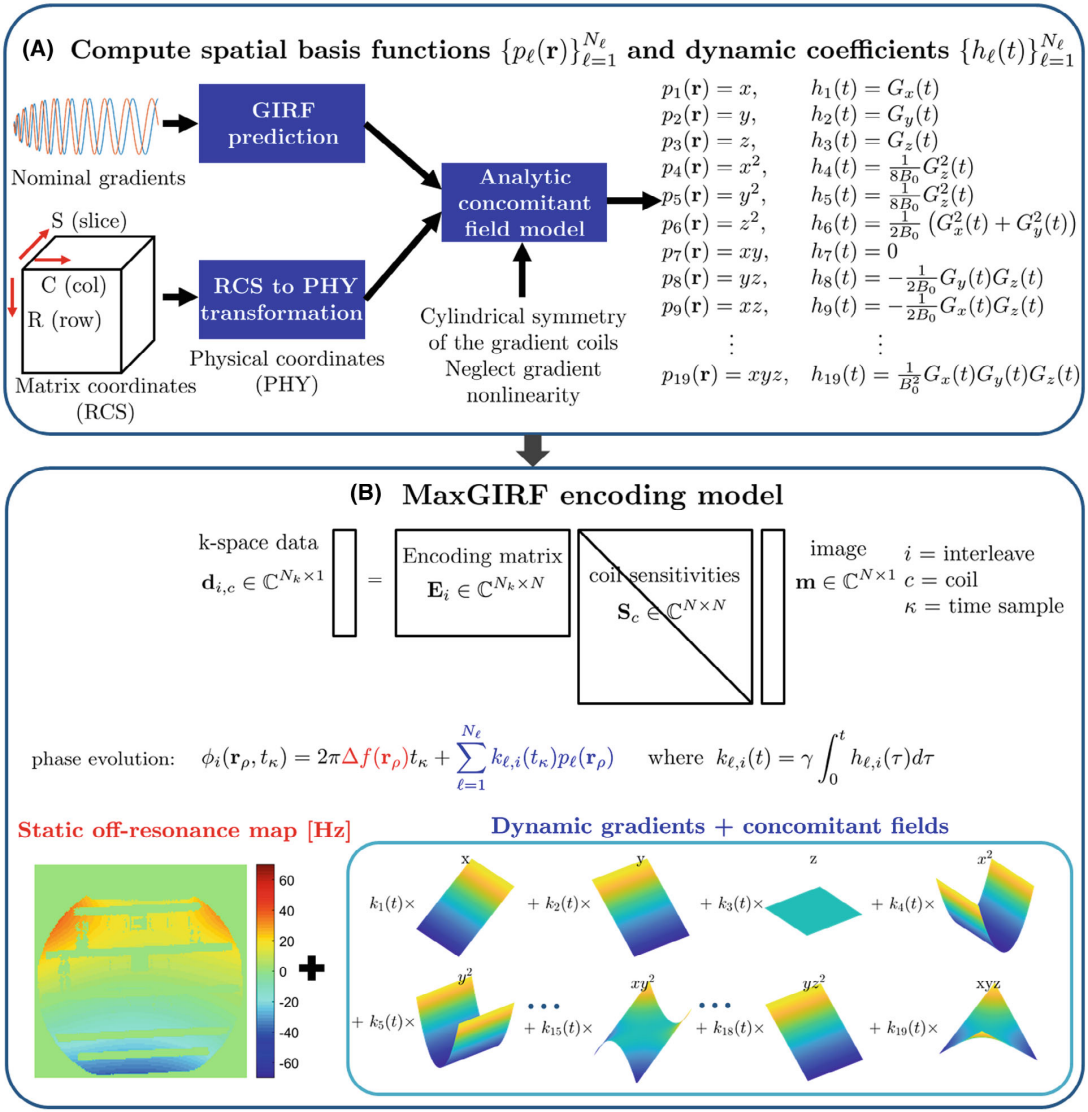
MaxGIRF reconstruction flowchart. A, Computation of concomitant field model: Gradient waveforms in the logical coordinate system are first transformed into the physical coordinate system. Distorted gradients in the physical coordinate system are estimated by gradient impulse response functions (GIRFs). Analytic expressions of concomitant fields derived from the coil geometry, presumed gradient nonlinearity, and GIRF‐predicted gradients, are calculated for each spatial position in the physical coordinate system. B, Encoding model: The MaxGIRF encoding model is an extension of the SENSE model that additionally includes phase terms due to static off‐resonance and concomitant fields. The phase evolution per voxel is represented as the sum of phase contributions from static off‐resonance (red) and spatial basis functions (blue) that include both linear gradients and concomitant field terms

Figure 1 illustrates the overall steps to calculate MaxGIRF encoding matrices. Let $G_{L}\left(t\right)$ and $G_{P}\left(t\right)$ be the gradients in the logical coordinate system and physical coordinate system, respectively. Unless clearly specified, we use the physical coordinate system exclusively and describe variables without the subscript for clarity (eg, $G\left(t\right)=G_{P}\left(t\right)$). Using a modified version of the expanded signal model,^10^, ^23^, ^36^ the measured k‐space data over the region‐of‐interest V is expressed as

(1) $$d_{i,c}\left(t\right)=\int_{V}m\left(r\right)S_{c}\left(r\right)\exp\left(-j\phi_{i}\left(r,t\right)\right)dr+n_{i,c}\left(t\right),$$

where $d_{i,c}$ denotes the ith interleaf, cth receive coil k‐space data of the target image m(r); $S_{c}\left(r\right)$ is the receive coil sensitivity at position r of the cth coil; $\phi_{i}\left(r,t\right)$ is the time‐varying phase of a voxel at position r in radians; and $n_{i,c}$ denotes the measurement noise. The indices i and c count the $N_{i}$ interleaves and $N_{c}$ receive coils, respectively. The MaxGIRF approach models the magnitude of the spatiotemporal magnetic field ${\left\|B_{i}\left(r,t\right)\right\|}_{\ell_{2}}$ as a sum of gradients $G_{i}\left(t\right)={\left[G_{x,i}\left(t\right),,,G_{y,i}\left(t\right),,,G_{z,i}\left(t\right)\right]}^{T}$ in millitesla per meter, static off‐resonance ∆f(r) in hertz, and concomitant fields in tesla^6^, as follows:

(2) $${\left\|B_{i}\left(r,t\right)\right\|}_{\ell_{2}}=B_{0}+G_{i}\left(t\right)\cdot r+2\pi \Delta f\left(r\right)/\gamma +\sum_{\ell =4}^{N_{\ell }}h_{\ell ,i}\left(t\right)p_{\ell }\left(r\right),$$

where ℓ counts the $N_{\ell }$ concomitant field terms; $p_{\ell }$ is the ℓth concomitant field basis function (in squared meters or cubed meters); and $h_{\ell ,i}$ is the ℓth dynamic coefficient (in Tesla squared meters or Tesla cubed meters), expressed as a function of the ith gradient waveforms; and γ is the gyromagnetic ratio (in radians per second per Tesla). Analytic expressions of ${\left\{h_{\ell ,i}\left(t\right)\right\}}_{\ell =1}^{N_{\ell }}$ and ${\left\{p_{\ell }\left(r\right)\right\}}_{\ell =1}^{N_{\ell }}$ for a symmetric gradient system used in this study^6^ are given in Table 1. The linear gradients are described as the first three terms in the concomitant field basis functions. Note that linear gradients $G_{i}\left(t\right)$ can be either GIRF‐predicted gradients $G_{i}^{\text{pred}}\left(t\right)\;$or nominal gradients $G_{i}^{\mathrm{nom}}\left(t\right)$. Time integration of the magnetic field (after the demodulation of its carrier frequency) multiplied by the gyromagnetic ratio γ, $\gamma \int_{0}^{t}{\left\|B_{i}\left(r,\tau \right)\right\|}_{\ell_{2}}d\tau$, then gives the phase evolution of a voxel at position r as follows:

(3) $$\begin{array}{cc} \phi_{i}\left(r,t\right) & =k_{i}\left(t\right)\cdot r+2\pi \Delta f\left(r\right)t+\sum_{\ell =4}^{N_{\ell }}k_{\ell ,i}\left(t\right)p_{\ell }\left(r\right),\\ & =k_{i}\left(t\right)\cdot r+{\tilde{\phi }}_{i}\left(r,t\right), \end{array}$$

where $k_{\ell ,i}\left(t\right)$ is the ℓth phase coefficient obtained by $k_{\ell ,i}\left(t\right)=\gamma \int_{0}^{t}h_{\ell ,i}\left(\tau \right)d\tau$; and $\tilde{\phi_{i}}\left(r,t\right)$ denotes a phase term consisting of static off‐resonance and concomitant fields. Note that the reference time point starts at the isodelay of an RF pulse for gradient‐echo pulse sequences and the TE for spin‐echo pulse sequences when spiral readouts start at TE. Let $N_{k}$ denote the number of k‐space samples per interleaf. Let $R_{\text{LtoP}}$ be a 3 × 3 orthogonal transformation matrix from the logical coordinate system to the physical coordinate system. Note that ${\left(R_{\text{LtoP}}\right)}^{T}={\left(R_{\text{LtoP}}\right)}^{-1}=R_{\text{PtoL}}$. Then we obtain

(4a) $$k_{P,i}\left(t\right)=R_{\text{LtoP}}k_{L,i}\left(t\right)$$

(4b) $$r_{P}=R_{\text{LtoP}}r_{L}+r_{P,\text{offset}}$$

where $r_{P,\text{offset}}$ represents the offset of a scan plane from isocenter in the physical coordinate system. With Eqs. 4a and 4b, we can express the k‐space phase $k_{i}\left(t\right)\cdot r$ in terms of variables in the logical coordinate system as follows:

(5) $$\begin{array}{cc} k_{P,i}\left(t\right)\cdot r_{P} & =k_{P,i}\left(t\right)\cdot \left(R_{\text{LtoP}}r_{L}+r_{P,\text{offset}}\right)\\ & \;={\left(R_{\text{LtoP}}k_{L,i}\left(t\right)\right)}^{T}R_{\text{LtoP}}r_{L}+k_{P,i}\left(t\right)\cdot r_{P,\text{offset}}\\ & =k_{L,i}\left(t\right)\cdot r_{L}+k_{P,i}\left(t\right)\cdot r_{P,\text{offset}}. \end{array}$$

The received signal can be expressed using variables both in the logical and physical coordinate systems, as follows:

(6) $$\begin{array}{cc} d_{i,c}\left(t\right) & =\int_{V}m\left(r\right)S_{c}\left(r\right)\exp\left(-jk_{P,i}\left(t\right)\cdot r_{P}\right)\\ & \;\times \exp\left(-j{\tilde{\phi }}_{i}\left(r_{P},,,t\right)\right)dr+n_{i,c}\left(t\right)\\ & =\exp\left(-jk_{P,i}\left(t\right)\cdot r_{P,\text{offset}}\right)\times \cdots \int_{V}m\left(r\right)S_{c}\left(r\right)\\ & \;\times \exp\;\left(-jk_{L,i}\left(t\right)\cdot r_{L}\right)\exp\;\left(\;-j{\tilde{\phi }}_{i}\left(r_{P},,,t\right)\right)dr+n_{i,c}\left(t\right). \end{array}$$

Equation 6 indicates that measured k‐space data are modulated by a time‐varying phase term due to a slice offset. If this time‐varying phase term is not compensated during data acquisition,^37^ then the received signal must be demodulated first before further processing, because concomitant field correction would not be accurate when voxels are displaced from their true locations. Note that a Fourier matrix is computed with the gradients in the logical coordinate system, as done in conventional fast Fourier transforms (FFT)/nonuniform fast Fourier transform (NUFFT), and a higher‐order encoding matrix is computed with k‐space trajectories and spatial coordinates in the physical coordinate system. See Supporting Information Text S1 for details about the coordinate transformations.

**TABLE 1:** Concomitant field basis functions ${\left\{p_{\ell }\left(r\right)\right\}}_{\ell =1}^{N_{\ell }}$ and dynamic coefficients ${\left\{h_{\ell ,i}\left(t\right)\right\}}_{\ell =1}^{N_{\ell }}$ for an MR system with symmetric gradient coils and zero gradient nonlinearity

Concomitant field basis functions	Dynamic coefficients	Type
$p_{1}\left(r\right)=x$	$h_{1,i}\left(t\right)=G_{x,i}\left(t\right)$	Gradient
$p_{2}\left(r\right)=y$	$h_{2,i}\left(t\right)=G_{y,i}\left(t\right)$	
$p_{3}\left(r\right)=z$	$h_{3,i}\left(t\right)=G_{z,i}\left(t\right)$	
$p_{4}\left(r\right)=x^{2}$	$h_{4,i}\left(t\right)=G_{z,i}^{2}\left(t\right)/\left(8B_{o}\right)$	Lowest order
$p_{5}\left(r\right)=y^{2}$	$h_{5,i}\left(t\right)=G_{z,i}^{2}\left(t\right)/\left(8B_{o}\right)$	
$p_{6}\left(r\right)=z^{2}$	$h_{6,i}\left(t\right)=\left(G_{x,i}^{2}\left(t\right)+G_{y,i}^{2}\left(t\right)\right)/\left(2B_{o}\right)$	
$p_{7}\left(r\right)=\mathrm{xy}$	$h_{7,i}\left(t\right)=0$	
$p_{8}\left(r\right)=\mathrm{yz}$	$h_{8,i}\left(t\right)=-G_{y,i}\left(t\right)G_{z,i}\left(t\right)/\left(2B_{o}\right)$	
$p_{9}\left(r\right)=\mathrm{xz}$	$h_{9,i}\left(t\right)=-G_{x,i}\left(t\right)G_{z,i}\left(t\right)/\left(2B_{o}\right)$	
$p_{10}\left(r\right)=x^{3}$	$h_{10,i}\left(t\right)=-\left(G_{x,i}\left(t\right)G_{z,i}^{2}\left(t\right)\right)/\left(8B_{o}^{2}\right)$	$1/B_{o}^{2}$ order
$p_{11}\left(r\right)=y^{3}$	$h_{11,i}\left(t\right)=-\left(G_{y,i}\left(t\right)G_{z,i}^{2}\left(t\right)\right)/\left(8B_{o}^{2}\right)$	
$p_{12}\left(r\right)=z^{3}$	$h_{12,i}\left(t\right)=-\left(G_{z,i}\left(t\right)\left(G_{x,i}^{2}\left(t\right)+G_{y,i}^{2}\left(t\right)\right)\right)/\left(2B_{o}^{2}\right)$	
$p_{13}\left(r\right)=x^{2}y$	$h_{13,i}\left(t\right)=-\left(G_{y,i}\left(t\right)G_{z,i}^{2}\left(t\right)\right)/\left(8B_{o}^{2}\right)$	
$p_{14}\left(r\right)=x^{2}z$	$h_{14,i}\left(t\right)=-\left(G_{z,i}^{3}\left(t\right)/4-G_{x,i}^{2}\left(t\right)G_{z,i}\left(t\right)\right)/\left(2B_{o}^{2}\right)$	
$p_{15}\left(r\right)=xy^{2}$	$h_{15,i}\left(t\right)=-\left(G_{x,i}\left(t\right)G_{z,i}^{2}\left(t\right)\right)/\left(8B_{o}^{2}\right)$	
$p_{16}\left(r\right)=y^{2}z$	$h_{16,i}\left(t\right)=-\left(G_{z,i}^{3}\left(t\right)/4-G_{y,i}^{2}\left(t\right)G_{z,i}\left(t\right)\right)/\left(2B_{o}^{2}\right)$	
$p_{17}\left(r\right)=xz^{2}$	$h_{17,i}\left(t\right)=-\left(G_{x,i}\left(t\right)\left(G_{x,i}^{2}\left(t\right)+G_{y,i}^{2}\left(t\right)\right)-G_{x,i}\left(t\right)G_{z,i}^{2}\left(t\right)\right)/\left(2B_{o}^{2}\right)$	
$p_{18}\left(r\right)=yz^{2}$	$h_{18,i}\left(t\right)=-\left(G_{y,i}\left(t\right)\left(G_{x,i}^{2}\left(t\right)+G_{y,i}^{2}\left(t\right)\right)-G_{y,i}\left(t\right)G_{z,i}^{2}\left(t\right)\right)/\left(2B_{o}^{2}\right)$	
$p_{19}\left(r\right)=\mathrm{xyz}$	$h_{19,i}\left(t\right)=\left(G_{x,i}\left(t\right)G_{y,i}\left(t\right)G_{z,i}\left(t\right)\right)/B_{o}^{2}$	

Suppose that an underlying object can be represented as a weighted sum of N ideal voxel shapes defined on an $N_{1}\times N_{2}$ Cartesian grid (ie, $m\left(r\right)=\sum_{\rho =1}^{N}m\left(r_{\rho }\right)\delta \left(r-r_{\rho }\right)$). Inserting this representation into Eq. 1 and discretizing in time leads to

(7) $$d_{i,c}=E_{i}S_{c}m+n_{i,c},$$

where $d_{i,c}={\left[d_{i,c}\left(t_{1}\right),,,\ldots ,,,d_{i,c}\left(t_{N_{k}}\right)\right]}^{T}\in {\mathbb{C}}^{N_{k}}$ contains the ith interleaf, cth coil (demodulated) k‐space data; $E_{i}\in {\mathbb{C}}^{N_{k}\times N}$ denotes the ith encoding matrix; $S_{c}\in {\mathbb{C}}^{N\times N}$ is a diagonal matrix containing the receive coil sensitivities of the cth coil; $m={\left[m\left(r_{1}\right),,,\ldots ,,,m\left(r_{N}\right)\right]}^{T}\in {\mathbb{C}}^{N}$ is a vector of complex image values; and $n_{i,c}={\left[n_{i,c}\left(t_{1}\right),,,\ldots ,,,n_{i,c}\left(t_{N_{k}}\right)\right]}^{T}\in {\mathbb{C}}^{N_{k}}$ contains the ith interleaf, cth coil measurement noise. The ith encoding matrix $E_{i}$ is expressed as the Hadamard product (element‐wise multiplication, denoted as ⊙) of a Fourier matrix $F_{i}\in {\mathbb{C}}^{N_{k}\times N}$ containing only linear phase terms and a higher‐order encoding matrix $H_{i}\in {\mathbb{C}}^{N_{k}\times N}$ containing other remaining phase terms:

(8) $$E_{i}=F_{i}⊙H_{i},$$

where

(9) $$F_{i}=\left[\begin{array}{ccc} \exp\left(-jk_{L,i}\left(t_{1}\right)\cdot r_{L,1}\right) & \cdots & \exp\left(-jk_{L,i}\left(t_{1}\right)\cdot r_{L,N}\right)\\ \vdots & \ddots & \vdots \\ \exp\left(-jk_{L,i}\left(t_{N_{k}}\right)\cdot r_{L,1}\right) & \cdots & \exp\left(-jk_{L,i}\left(t_{N_{k}}\right)\cdot r_{L,N}\right) \end{array}\right],$$

and

(10) $$H_{i}=\left[\begin{array}{ccc} \exp\left(-j{\tilde{\phi }}_{i}\left(r_{P,1},,,t_{1}\right)\right) & \cdots & \exp\left(-j{\tilde{\phi }}_{i}\left(r_{P,N},,,t_{1}\right)\right)\\ \vdots & \ddots & \vdots \\ \exp\left(-j{\tilde{\phi }}_{i}\left(r_{P,1},,,t_{N_{k}}\right)\right) & \cdots & \exp\left(-j{\tilde{\phi }}_{i}\left(r_{P,N},,,t_{N_{k}}\right)\right) \end{array}\right].$$

It is important to note that the forward signal model in Eq. 1 is described with the forward Fourier transform as commonly done in standard textbooks, but the choice of FFT versus inverse FFT for transforming k‐space data to an image that is vendor‐specific, and critical for a successful implementation.^7^, ^38^

### 2.2 Image reconstruction

Image reconstruction for MaxGIRF encoding can be formulated as a linear least‐squares problem similar to Refs 10, 13, 36. Specifically, the MaxGIRF approach uses a multishot extension of Ref 10, as follows:

(11) $$\hat{m}=\underset{m}{\text{argmin}}\sum_{i=1}^{N_{i}}\sum_{c=1}^{N_{c}}{\left\|d_{i,c}-E_{i}S_{c}m\right\|}_{\ell_{2}}^{2}.$$

Equation 11 often needs to be expressed in the form of A(m)=b to be solved with iterative algorithms (eg, LSQR^39^). Such a form is obtained by taking the derivative of a cost function with respect to m and setting it equal to zero, as follows:

(12) $$\sum_{i=1}^{N_{i}}\sum_{c=1}^{N_{c}}\left(A_{i,c}^{H}A_{i,c}\right)m=\sum_{i=1}^{N_{i}}\sum_{c=1}^{N_{c}}A_{i,c}^{H}\left(d_{i,c}\right),$$

where $A_{i,c}\left(x\right)=E_{i}S_{c}x:{\mathbb{C}}^{N}\to {\mathbb{C}}^{N_{k}}$ denotes the linear forward operator that maps a length‐N vector of image values to a length‐$N_{k}$ vector of k‐space samples of the ith interleaf and cth coil; and $A_{i,c}^{H}\left(y\right)=S_{c}^{H}E_{i}^{H}y:{\mathbb{C}}^{N_{k}}\to {\mathbb{C}}^{N}$ denotes its adjoint. The superscript ${\left(\cdot \right)}^{H}$ denotes the transposed complex conjugate.

### 2.3 Low‐rank approximation to higher‐order encoding matrices

To reduce the computational burden of explicit matrix–vector multiplications and reduce memory requirements, we introduce a low‐rank approximation to higher‐order encoding matrices following the previous approaches.^40^, ^41^ Suppose the singular value decomposition (SVD) of the ith higher‐order encoding matrix $H_{i}\in {\mathbb{C}}^{N_{k}\times N}$ is given by

(13) $$H_{i}=\sum_{\ell =1}^{L_{\max}}u_{\ell ,i}\sigma_{\ell ,i}{\tilde{v}}_{\ell ,i}^{H}=\sum_{\ell =1}^{L_{\max}}u_{\ell ,i}v_{\ell ,i}^{H},$$

where $u_{\ell ,i}\in {\mathbb{C}}^{N_{k}}$ denotes the ℓth left singular vector; $\sigma_{\ell ,i}\in \mathbb{R}$ is theℓth singular value; ${\tilde{v}}_{\ell ,i}\in {\mathbb{C}}^{N}$ is the ℓth right singular vector; and $L_{\max}$ denotes the true rank of the higher‐order encoding matrix $H_{i}$. A singular value and the corresponding right singular vector can be combined to yield $v_{\ell ,i}\in {\mathbb{C}}^{N}$. The vectors $u_{\ell ,i}\in {\mathbb{C}}^{N_{k}}$ and $v_{\ell ,i}\in {\mathbb{C}}^{N}$ are hereafter referred to as temporal and spatial basis vectors for the ith higher‐order encoding matrix $H_{i}$, respectively. Note that the relation in Eq. 13 is exact (no loss in accuracy), and $L_{\max}$ is large (> 50) in general. According to the Eckart‐Young theorem,^42^ the rank‐L SVD truncation ${\tilde{H}}_{i}=\sum_{\ell =1}^{L}u_{\ell ,i}v_{\ell ,i}^{H}$ provides the best rank‐L approximation to $H_{i}$ in a least‐squares sense, as follows:

(14) $${\left\|H_{i}-{\tilde{H}}_{i}\right\|}_{F}=\underset{\mathrm{rank}\left(B\right)\leq L}{\text{argmin}}{\left\|H_{i}-B\right\|}_{F}=\sqrt{\sigma_{L+1}^{2}+\cdots +\sigma_{L\max}^{2}}.$$

We select only one L and apply it to all higher‐order encoding matrices. Substituting ${\tilde{H}}_{i}=\sum_{\ell =1}^{L}u_{\ell ,i}v_{\ell ,i}^{H}$ into Eq. 8 yields

$$E_{i}\approx F_{i}⊙\left(\sum_{\ell =1}^{L}u_{\ell ,i}v_{\ell ,i}^{H}\right)\;$$

$$\approx \sum_{\ell =1}^{L}F_{i}⊙\left(u_{\ell ,i}v_{\ell ,i}^{H}\right)\;$$

(15) $$\approx \sum_{\ell =1}^{L}\mathrm{diag}\left(u_{\ell ,i}\right)F_{i}\mathrm{diag}\left(v_{\ell ,i}^{*}\right),$$

where $\mathrm{diag}\left(u_{\ell ,i}\right)\in {\mathbb{C}}^{N_{k}\times N_{k}}$ and $\mathrm{diag}\left(v_{\ell ,i}^{*}\right)\in {\mathbb{C}}^{N\times N}$ are diagonal matrices containing the elements of the vectors $u_{\ell ,i}$ and $v_{\ell ,i}^{*}$ (the complex conjugate of $v_{\ell ,i})$ in the main diagonal, respectively. The last expression is obtained using the special property of the Hadamard product of a dense matrix $F_{i}$ with a rank‐1 matrix $u_{\ell ,i}v_{\ell ,i}^{H}$. Using Eq. 15, the forward and adjoint operators can be expressed as

(16a) $$A_{i,c}\left(x\right)=E_{i}S_{c}x\approx \sum_{\ell =1}^{L}\mathrm{diag}\left(u_{\ell ,i}\right)F_{i}\mathrm{diag}\left(v_{\ell ,i}^{*}\right)S_{c}x,$$

(16b) $$A_{i,c}^{H}\left(y\right)=S_{c}^{H}E_{i}^{H}y\approx S_{c}^{H}\sum_{\ell =1}^{L}\mathrm{diag}\left(v_{\ell ,i}\right)F_{i}^{H}\mathrm{diag}\left(u_{\ell ,i}^{*}\right)y.$$

Equation 16indicates that an expensive, explicit matrix–vector multiplication with an encoding matrix $E_{i}$ (and $E_{i}^{H})$ can be replaced by L summations of a fast routine for $F_{i}$, such as FFT followed by inverse gridding^43^ or NUFFT.^44^

### 2.4 Static off‐resonance map estimation

The MaxGIRF reconstruction requires an accurate and spatially smooth static off‐resonance map. For this purpose, we acquire a series of Cartesian gradient‐echo data sets at different TEs. Because the MaxGIRF encoding model does not separate water/fat components, we consider the image content $\rho ={\left[\rho \left(r_{1}\right),,,\ldots ,,,\rho \left(r_{N}\right)\right]}^{T}\in {\mathbb{C}}^{N}$ as a sum of water/fat, and model static off‐resonance $\mathrm{\Delta f}={\left[\Delta f\left(r_{1}\right),,,\ldots ,,,\Delta f\left(r_{N}\right)\right]}^{T}\in {\mathbb{R}}^{N}$ (in Hertz) as a sum of $B_{0}$ inhomogeneity and the water/fat chemical shift (eg, −3.8 ppm, −88 Hz at 0.55 T). We perform image‐based parameter estimation using nonlinear inversion optimization, inspired by a recent work on water/fat separation and $B_{0}$ inhomogeneity mapping.^45^, ^46^ Specifically, the forward signal model is defined as

(17) $$\begin{array}{cc} F_{m}\left(x\right) & =\rho ⊙\exp\left(j2\pi \mathrm{\Delta f}{\mathrm{TE}}_{m}\right)\\ & =\left[\begin{array}{c} \rho \left(r_{1}\right)\\ \vdots \\ \rho \left(r_{N}\right) \end{array}\right]⊙\left[\begin{array}{c} \exp\left(j2\pi \Delta f\left(r_{1}\right){\mathrm{TE}}_{m}\right)\\ \vdots \\ \exp\left(j2\pi \Delta f\left(r_{N}\right){\mathrm{TE}}_{m}\right) \end{array}\right]\\ & =\mathrm{diag}\left(\exp\left(j2\pi \mathrm{\Delta f}{\mathrm{TE}}_{m}\right)\right)\rho \\ & =\mathrm{diag}\left(\rho \right)\exp\left(j2\pi \mathrm{\Delta f}{\mathrm{TE}}_{m}\right)\\ & \;\text{with unknown}\;x={\left[\rho^{T},,,{\mathrm{\Delta f}}^{T}\right]}^{T}\\ & \;\text{and}\;m=1,\ldots N_{e}, \end{array}$$

where $F_{m}\left(x\right)\in {\mathbb{C}}^{N}$ is a length‐N vector of the estimated mth TE image; $N_{e}$ denotes the number of TEs; ${\mathrm{TE}}_{m}$ is the mth TE in seconds; and the symbol ⊙ denotes the Hadamard product. Equation 17 is solved with the a slight modification of the iteratively regularized Gauss‐Newton method, as described by Tan et al.^45^ The modified cost function is given as

$$\Phi \left(\hat{x}\right)=\underset{\hat{x}}{\text{argmin}}\|{y-G\left(\hat{x}\right)\|}_{\ell_{2}}^{2}+\alpha .\|\hat{x}-{\hat{x}}_{0}{\|}_{\ell_{2}}^{2}$$

(18) $$\;\text{with}\;x=W\hat{x}\;\text{and}\;G\left(\hat{x}\right)=F\left(W\hat{x}\right),$$

where $y\in {\mathbb{C}}^{N_{e}N\times 1}$ is a length‐$N_{e}N$ vector of the concatenation of all noisy reconstructed echo images $F\left(x\right)={\left[F_{1}{\left(x\right)}^{T},,,\ldots ,,,F_{N_{e}}{\left(x\right)}^{T}\right]}^{T}\in {\mathbb{C}}^{N_{e}N\times 1}$; α is the regularization parameter; and ${\hat{x}}_{0}$ is a starting initial guess. A preconditioning matrix $W\in {\mathbb{C}}^{2N\times 2N}$ contains a Sobolev norm that enforces spatial smoothness on the static off‐resonance map as follows:

(19) $$\begin{array}{cc} & \left[\begin{array}{c} \rho \\ \mathrm{\Delta f} \end{array}\right]=\left[\begin{array}{cc} I_{N} & 0\\ 0 & F^{-1}{\left(1+w{\left\|k\right\|}_{\ell_{2}}^{2}\right)}^{-h} \end{array}\right]\left[\begin{array}{c} \hat{\rho }\\ \hat{\mathrm{\Delta f}} \end{array}\right],\\ & \;\times {\left(1+w{\left\|k\right\|}_{\ell_{2}}^{2}\right)}^{-h}\hat{\mathrm{\Delta f}}\\ & ≜\left[\begin{array}{ccc} {\left(1+w{\left\|{\vec{k}}_{1}\right\|}_{\ell_{2}}^{2}\right)}^{-h} & 0 & 0\\ 0 & \ddots & 0\\ 0 & 0 & {\left(1+w{\left\|{\vec{k}}_{N}\right\|}_{\ell_{2}}^{2}\right)}^{-h} \end{array}\right]\left[\begin{array}{c} {\hat{\Delta f}}_{1}\\ \vdots \\ {\hat{\Delta f}}_{N} \end{array}\right], \end{array}$$

where $I_{N}\in {\mathbb{R}}^{N\times N}$ is an identity matrix; $F^{-1}\in {\mathbb{C}}^{N\times N}$ is a unitary 2D inverse Fourier transform matrix; $\vec{k}$ $\in {\mathbb{R}}^{2\times 1}$ is normalized Cartesian k‐space coordinates defined in [−0.5,0.5]×[−0.5,0.5]; and w,h∈ℝ are constants set to 32 and 16, respectively. Equation 18 is solved with the iteratively regularized Gauss‐Newton method (see Appendix).

## 3 METHODS

### 3.1 Reconstruction and image processing

Cartesian and spiral‐image reconstructions and postprocessing were performed in *MATLAB* R2020b (MathWorks, Natick, MA) on a PC equipped with one 1.60‐GHz 4‐core Intel i5‐8250U CPU and 20 GB of RAM. A vendor proprietary raw data format was converted into the ISMRMRD format^47^ and read in *MATLAB*.^48^ For both Cartesian and spiral reconstructions, FFT was applied to transform from k‐space to image space. Coil sensitivity maps were estimated using the Walsh method^49^ from the 32 × 32 Hanning‐windowed center of k‐space data (gridded k‐space data for spiral acquisitions). Neither intensity normalization nor gradient nonlinearity correction were applied. Spiral trajectories were generated with Ref 50. A sample density compensation function^51^ was computed with Ref 52. The NUFFT code was downloaded from Ref 53. The MaxGIRF reconstructions were performed with the LSQR algorithm with maximum number of iterations = 15 and tolerance = 1e−5. For static off‐resonance map calculation, a coil sensitivity map from the first echo image was used to reconstruct coil‐combined images of the other echoes. A smooth static off‐resonance map was estimated by the iteratively regularized Gauss‐Newton (GN) method with $\alpha_{\min}=$ 1e−6,^54^ GN iterations = 35, maximum number of LSQR iterations = 250, and tolerance of LSQR = 1e−10.

### 3.2 Selection of an optimal rank L

We chose an optimal L that gives less than 2% error in normalized RMS error (NRMSE) between complex‐valued full‐rank and low‐rank reconstructions: $\text{NRMSE}={\left\|m_{\text{full}}-m_{\mathrm{low}}\right\|}_{\ell_{2}}/{\left\|m_{\text{full}}\right\|}_{\ell_{2}}$. In vivo multislice spiral spin‐echo axial and slightly oblique sagittal data sets were used for evaluation. A randomized SVD algorithm as described in Supporting Information Text S2 was used to compute the SVD of a higher‐order encoding matrix. Singular values up to 50/80 (axial/sagittal) were calculated and considered as full rank. Image reconstructions were performed with a conjugate phase reconstruction (ie, the right side of Eq. 12).

### 3.3 Numerical simulation

To validate the proposed MaxGIRF approach, noiseless simulations on brain images with simulated eight‐channel coil sensitivity maps, 256 × 256 matrix, were performed. A sagittal slice was obtained from a 3D MIDA (multimodal imaging–based detailed anatomical) brain phantom,^55^ and coil sensitivity maps were obtained from Ref 56. The 116 tissue types of a MIDA phantom were categorized into 13 tissue labels used in a Brainweb phantom^57^ by visual matching. The MR parameters ($T_{1}/T_{2}/T_{2}^{*}/M_{0}$) were obtained from a Brainweb phantom acquired at 1.5 T, and the dependence of relaxation parameters on the main magnetic field strength was ignored. A 20‐interleaf, variable‐density spiral acquisition (9.2‐ms readout) was simulated with $G_{\max}$= 24 mT/m, $S_{\max}$ = 144 T/m/s, ADC dwell time = 2.5 μs, resolution = 0.9375 × 0.9375 mm^2^, and FOV decreasing from 240 × 240 mm^2^ to 180 × 180 mm^2^. The base spiral interleaf was similar to that used in 3D brain MR fingerprinting.^58^ Direct matrix–vector multiplications using Eqs. 7 and 8 were used to generate noiseless k‐space data. System imperfections such as static off‐resonance and eddy currents were not simulated. The B_0_ dependence (0.55 T, 1.5 T, 3 T, and 7 T) and off‐isocenter dependence (*z* = 0, 50, 100, 150, and 200 mm) of concomitant fields were simulated. The MaxGIRF reconstructions were performed with a low‐rank approximation ($L/L_{\max}$=50/80) and NUFFT. The NRMSE between a Cartesian reference and spiral reconstructions was calculated. A time‐averaged concomitant field map for the first interleaf (in Hertz), $f_{c,1}\left(r\right)$, over the spiral readout duration (T) was calculated to demonstrate its relative magnitude compared with a static off‐resonance map^59^ as follows:

(21) $$\begin{array}{cc} f_{c,1}\left(r\right) & =\frac{1}{2\pi T}\sum_{\ell =4}^{N_{\ell }}\int_{0}^{T}h_{\ell ,1}\left(\tau \right)d\tau \;p_{\ell }\left(r\right)\\ & =\frac{1}{2\pi T}\sum_{\ell =4}^{N_{\ell }}k_{\ell ,1}\left(T\right)\;p_{\ell }\left(r\right). \end{array}$$

### 3.4 Imaging system

All imaging experiments were performed on a high‐performance 0.55T scanner (prototype MAGNETOM Aera; Siemens Healthcare, Erlangen, Germany) with gradients capable of 45 mT/m amplitude and 200 T/m/s slew rate.^34^, ^35^ A 16‐channel head/neck receive coil was used for phantom and in vivo experiments.

### 3.5 The GIRF measurements

The GIRF measurements were obtained using a set of triangular input functions and a spherical phantom as described by Campbell‐Washburn et al.^29^ A body coil was used for both RF transmission and signal reception. The Brodsky method^25^ was used to estimate both B_0_ cross‐terms and first‐order self‐term GIRFs as described by Robinson et al.^31^ Only self‐term GIRFs were used in this study.

### 3.6 Phantom experiments

Spiral scans (axial and sagittal) of an ISMRM/NIST system phantom were acquired with a 2D gradient‐echo pulse sequence. An 8‐interleaf, uniform‐density, spiral‐out trajectory was designed to have 11.8‐ms readout duration. A target axial slice was imaged at isocenter and 75‐mm off‐isocenter in the *z*‐direction. A sagittal slice was imaged at isocenter. Imaging parameters were FOV = 224 × 224 mm^2^, resolution = 1.4 × 1.4 mm^2^, slice thickness = 8 mm, flip angle = 20°, TR = 100 ms, TE = 1 ms, and number of signal averages = 1. Ten repetitions were performed to reach steady state. For a static off‐resonance map, a single‐echo 2D Cartesian gradient‐echo sequence was repeated to acquire data sets at different TEs (2.5, 3.7, 4.7, 5.7, 6.7, and 7.7 ms).

### 3.7 Human experiments

All volunteers were scanned under a protocol approved by our local institutional review board (clinicaltrials.gov NCT03331380) and provided written informed consent. In vivo human brain scans (axial and sagittal) were acquired with a 2D interleaved multislice spiral spin‐echo pulse sequence. A slice‐rephasing gradient and the left crusher of a refocusing pulse were combined with a waveform reshaping technique^60^ to minimize the concomitant‐field phase. Spoiler gradients were applied on all three axes at the end of a readout. Imaging parameters were FOV = 240 × 240 mm^2^, resolution = 0.75 × 0.75 mm^2^, slice thickness = 5 mm, slice gap = 15 mm, flip angle = 90°, TR = 745/500 (spiral/Cartesian) ms, TE = 15 ms, ADC dwell time = 2.5 μs, readout duration = 11.89 ms, number of readout samples = 4756, number of interleaves = 24, and number of signal averages = 14. For comparison, King's method was used for both axial and sagittal scans. Additionally, a modified King's method including static off‐resonance correction was performed for axial scans. Specifically, after correcting a time‐varying global frequency offset (through‐plane correction of concomitant field‐induced phase), frequency‐segmented deblurring was performed for in‐plane blurring correction, using an ordinary time parameter and a static off‐resonance map for Eqs. 26 and 30 in King et al,^7^ respectively.

## 4 RESULTS

**FIGURE 2:**
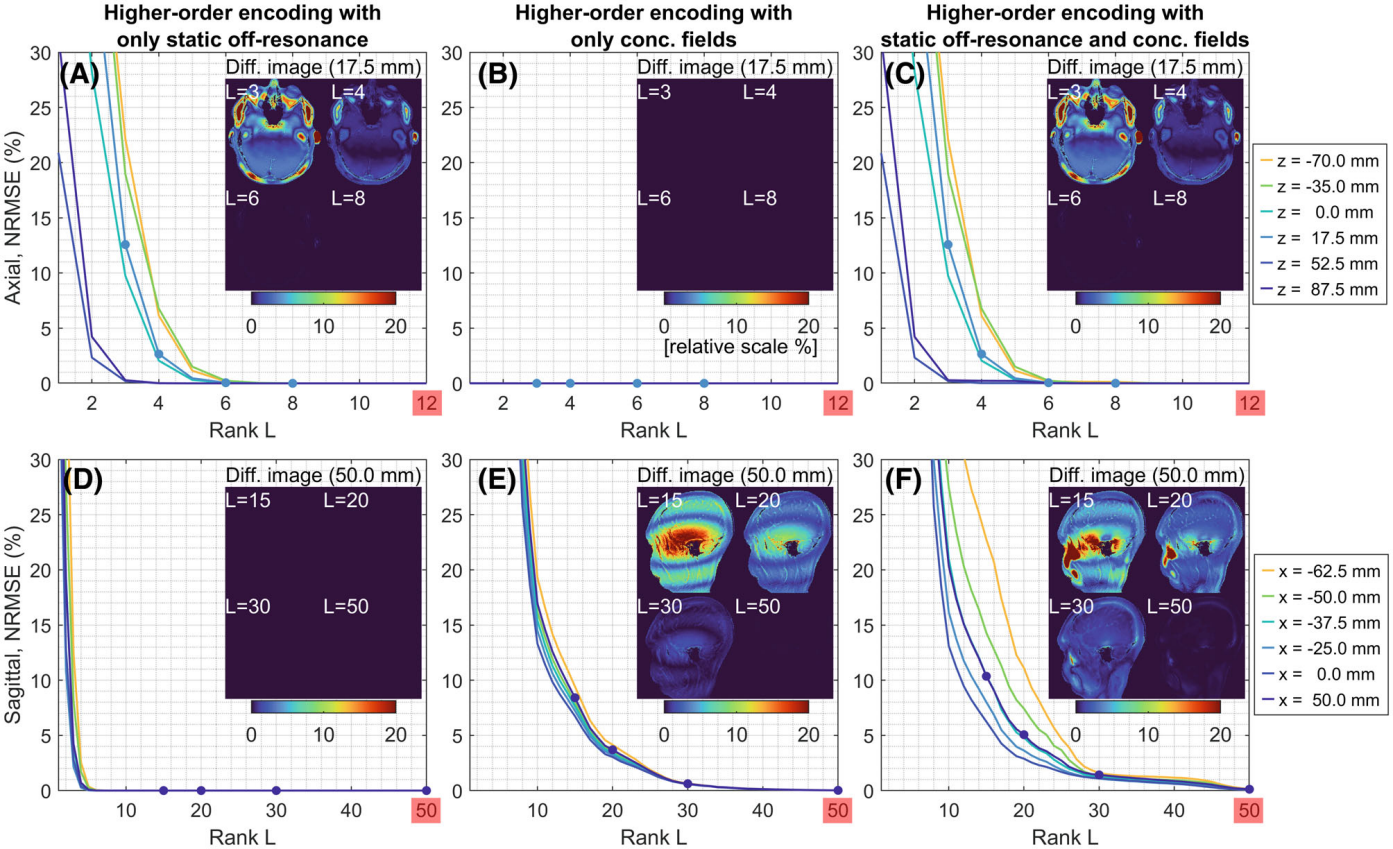
Low‐rank approximations of the MaxGIRF higher‐order encoding matrix are effective. Normalized RMS errors (NRMSEs) are measured between full‐rank image reconstructions and low‐rank approximations from in vivo multislice spiral spin‐echo axial and sagittal datasets. A–F, The NRMSEs when only static off‐resonance is included (A,D), only concomitant fields are included (B,E), both static off‐resonance and concomitant fields are included (C,F) in the higher‐order encoding matrices. The inset images show the difference between full‐rank (50/80 for axial/sagittal) and L‐rank reconstructions. Note that a different range of the *x*‐axis is used for clarity

Figure 2 shows the NRMSEs between full‐rank and low‐rank reconstructions from in vivo multislice spiral spin‐echo axial and sagittal data sets. The NRMSEs are provided as a function of rank L when only static off‐resonance is included (A,D), when only concomitant fields are included (B,E), and when both static off‐resonance and concomitant fields are included (C,F) in the higher‐order encoding matrices. For axial orientation, because the effect of concomitant fields is a time‐dependent receive frequency shift, its contribution to the rank is minimal (Figure 2B); thus, the static off‐resonance contributes mostly to the rank (Figure 2A). For nonaxial orientations, because the effect of concomitant fields is spatiotemporal blurring, a large rank is required compared with that in axial orientation. The rank of static off‐resonance is less than 8 like axial orientation and smaller than the rank of concomitant fields in absolute sense (Figure 2D vs 2E). The low‐rank ($L/L_{\max}$=8/50) reconstruction in Figure 2C gives almost perfect reconstruction for all axial slices, and the low‐rank ($L/L_{\max}$=30/80) reconstruction in Figure 2F gives < 2% error for all sagittal slices. The signal‐intensity attenuation is primarily in regions with high off‐resonance. The maximum deviation within the brain cortex of the difference between full‐rank and low‐rank (L= 30) reconstructions is < 2% for all sagittal slices (only a slice at *x* = 50.0 mm is shown). The reconstruction time for the noniterative, conjugate phase‐based MaxGIRF (also iterative MaxGIRF) is linearly scaled by the rank (ie, number of singular values). The reconstruction times per singular value for axial and sagittal orientations were 5 s and 8 s, respectively. Thus, the reconstruction times (low‐rank/full‐rank) for axial and sagittal orientations were 40/250 s (8/50 rank) and 240/640 s (30/80 rank), respectively.

**FIGURE 3:**
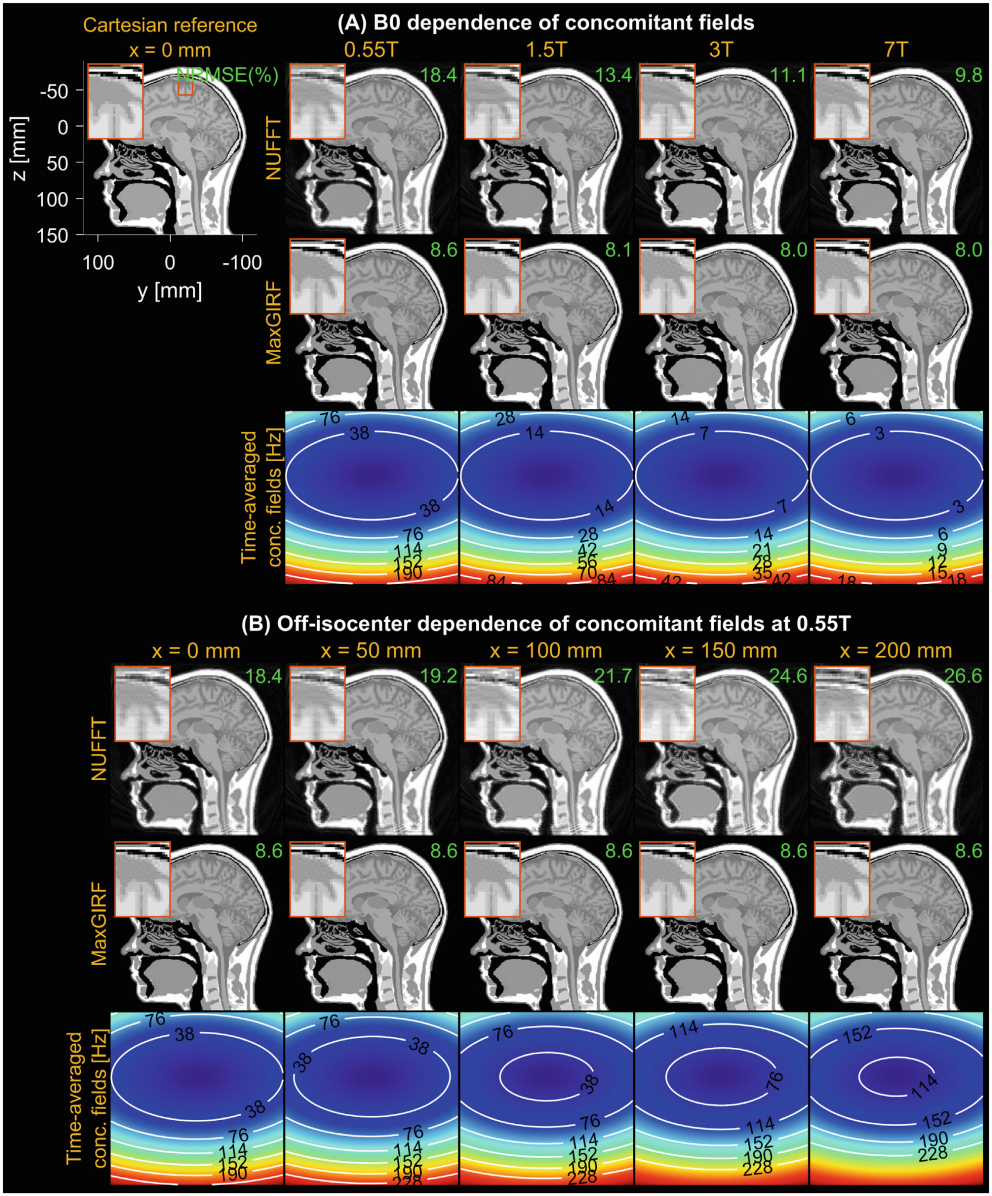
Evaluation of concomitant field correction using MaxGIRF reconstruction of noiseless numerical simulations. A, Dependence of concomitant fields on B_0_, using field strengths (0.55 T, 1.5 T, 3 T, and 7 T) at a slice position of 0 mm from isocenter. A reference image used to simulate non‐Cartesian k‐space data is shown along with the physical coordinate system. The NRMSE between the ground truth and spiral reconstruction is shown (green), with 8.6% (neglecting small changes at higher field strengths) being the minimum achievable error from the difference between Cartesian and spiral image reconstructions. B, Dependence of concomitant fields on off‐isocenter distance is demonstrated for sagittal prescription. A time‐averaged concomitant field map indicates the relative strength of concomitant fields at various B_0_ and distances from isocenter. Nonuniform fast Fourier transform (NUFFT) reconstruction shows increased spatial blurring as the field strength decreases and the distance from isocenter increases

Figure 3 demonstrates noiseless numerical simulations of MaxGIRF reconstruction, using a low‐rank approximation ($L/L_{\max}$ = 50/80). The NRMSEs for MaxGIRF at *x* = 0 mm decreased gradually from 8.6% to 8.0% as the field strength increases. This small decrease in NRMSEs is attributed to weaker concomitant fields at higher field strengths and did not make any noticeable difference in image quality. This minimum error (8.6%) is primarily caused by the difference between Cartesian and spiral image reconstructions. Application of MaxGIRF reconstruction on off‐isocenter acquisitions achieved this minimum error, indicating perfect correction of the concomitant fields.

**FIGURE 4:**
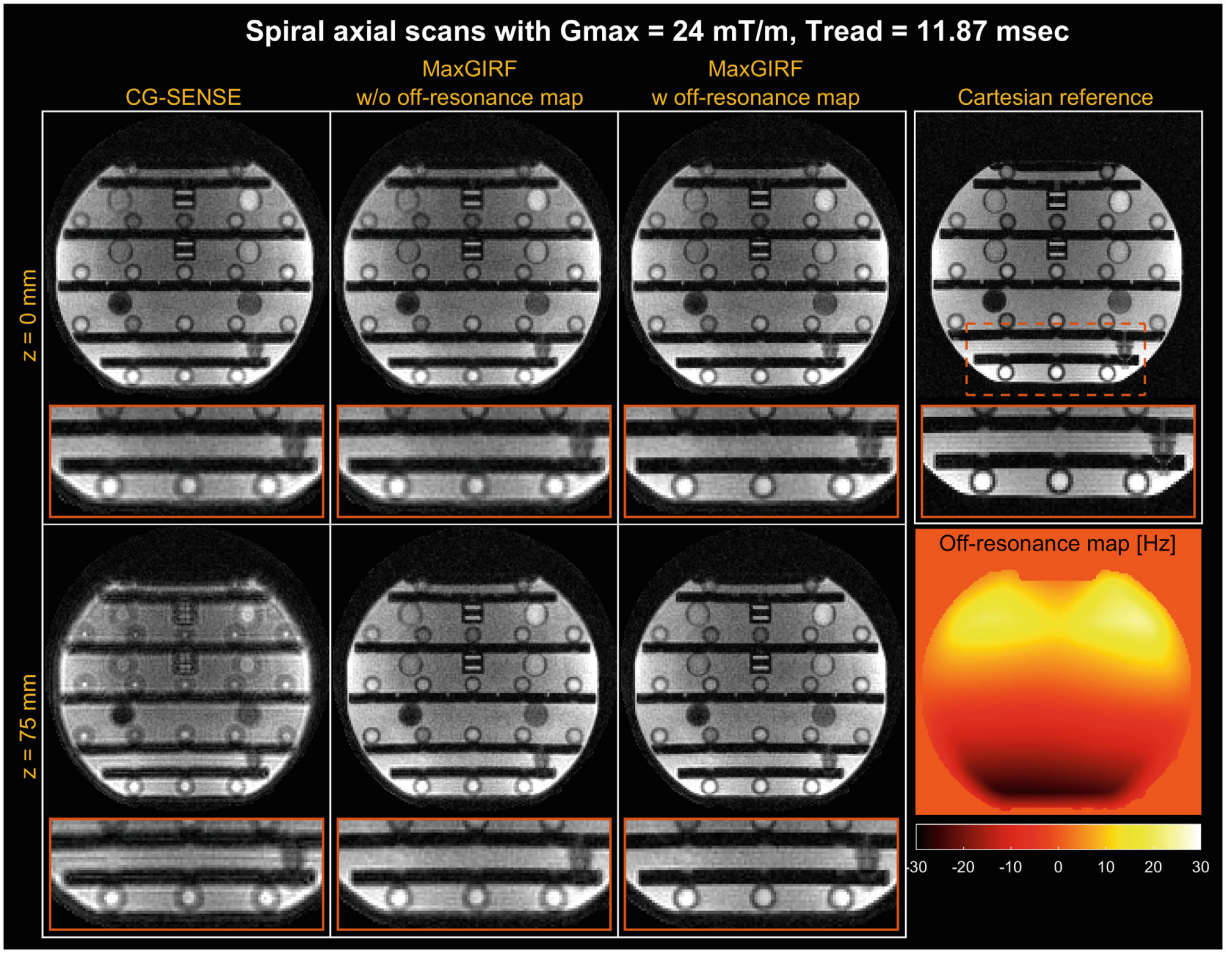
Spiral axial imaging of an International Society for Magnetic Resonance in Medicine/National Institute of Standards and Technology (ISMRM/NIST) phantom at 0.55 T. Top row: Isocenter. Bottom row: Off‐isocenter with *z* = 75 mm. A 2D Cartesian gradient‐echo (GRE) reference is also shown (TE and SNR are not matched). Conjugate gradient–based SENSE (CG‐SENSE; first column) clearly shows spatial blurring caused by both concomitant fields and static off‐resonance. The MaxGIRF reconstruction can be applied without (second column) and with (third column) a separately acquired static off‐resonance map. The MaxGIRF approach without a static off‐resonance map dramatically improves the image quality from CG‐SENSE, and further improvements are achieved with a static off‐resonance map (one exemplary region shown in the orange box)

Figure 4 shows MaxGIRF reconstruction (L = 8) on axial spiral scans of an ISMRM/NIST phantom at 0.55 T. The blurring caused by the static off‐resonance and concomitant fields is successfully removed as compared with the conventional conjugate gradient–based iterative SENSE (CG‐SENSE) reconstruction. The inclusion of a static off‐resonance map in MaxGIRF reconstruction further improves the sharpness of features in regions with nonzero off‐resonance.

**FIGURE 5:**
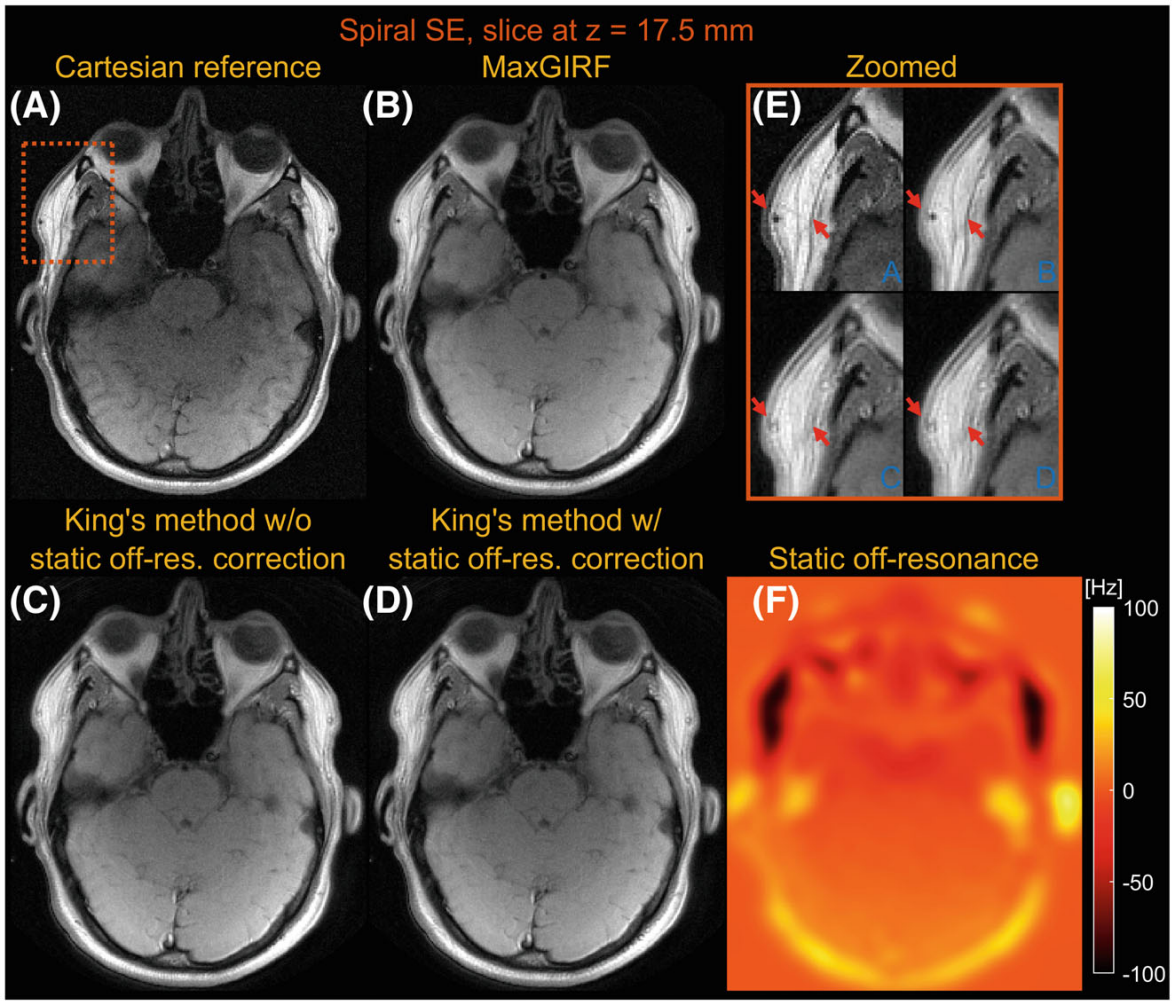
Axial spiral spin‐echo (SE) imaging of a healthy volunteer at 0.55 T close to isocenter (*z* = 17.5 mm). Comparison of image reconstructions using comparator Cartesian spin‐echo image (A), MaxGIRF reconstruction with static off‐resonance correction (Low‐rank approximation L = 8) (B), King's method without static off‐resonance correction (C), and King's method with static off‐resonance correction (D). E, Zoomed‐in image of a region with large static off‐resonance (orange box). F, Static off‐resonance map. King's method with static off‐resonance correction shows minor improvements compared to without static off‐resonance correction in regions with strong, sharply varying static off‐resonance. In contrast to both King's methods, MaxGIRF reconstruction successfully resolves local blurring due to strong off‐resonance and provides features comparable to the Cartesian spin‐echo image (eg, orange box)

**FIGURE 6:**
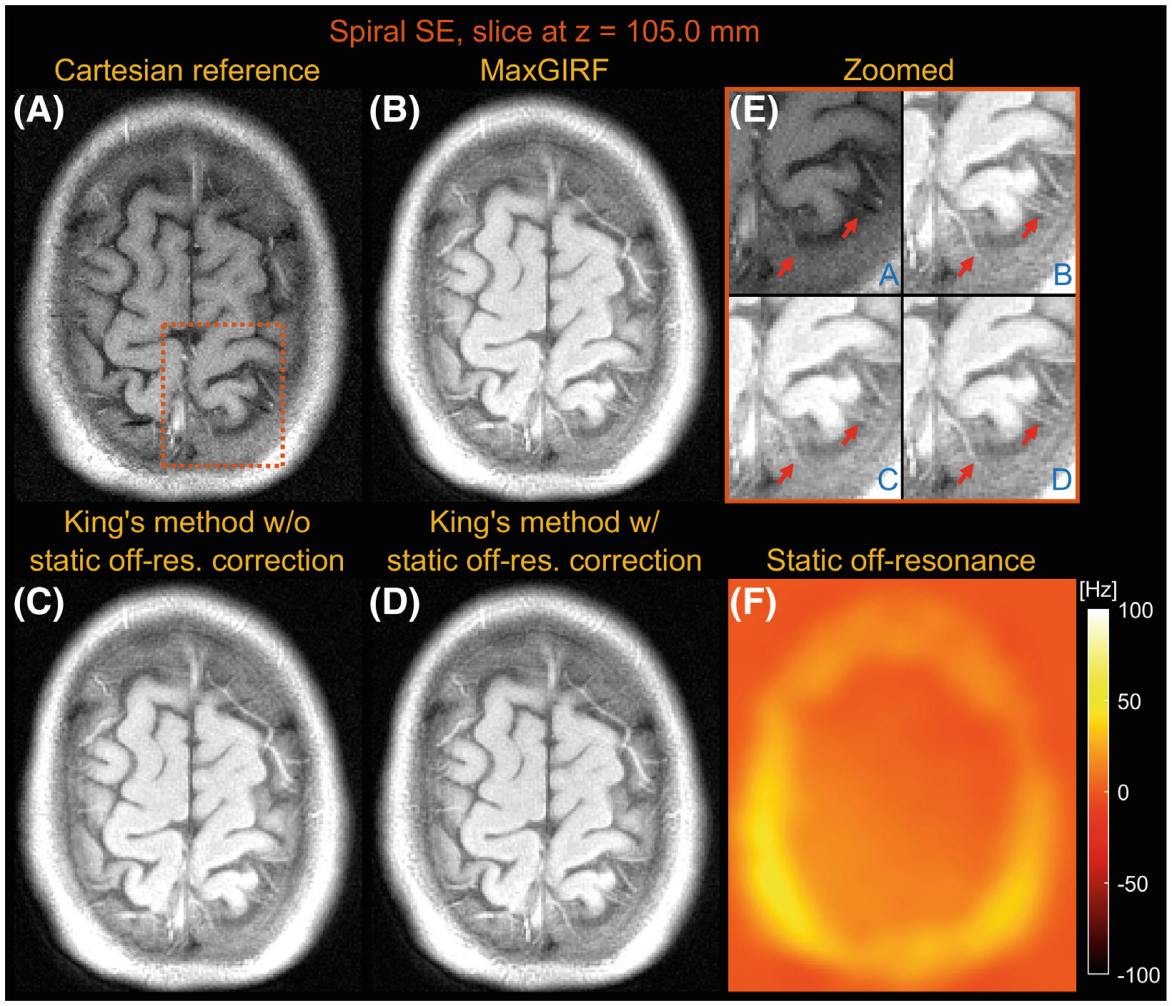
Axial spiral spin‐echo imaging of a healthy volunteer at 0.55 T far from isocenter (*z* = 105.0 mm). Comparison of image reconstructions using comparator Cartesian spin‐echo image (A), MaxGIRF reconstruction with static off‐resonance correction (Low‐rank approximation L = 8) (B), King's method without static off‐resonance correction (C), and King's method with static off‐resonance correction (D). E, Zoomed‐in image (orange box). F, Static off‐resonance map. For an axial slice without angulation, the effect of concomitant fields is merely a time‐varying global frequency offset; thus, all three methods successfully resolve spatial blurring due to concomitant fields when compared with NUFFT (not shown). In contrast to its performance in Figure 5, King's method with static off‐resonance correction performs well particularly in this slice, because a static off‐resonance map varies slowly in space, which is required for successful application of most noniterative off‐resonance correction methods. The MaxGIRF reconstruction based on iterative conjugate gradient (CG) shows improved delineation of tissue boundaries compared with King's method without static off‐resonance correction, regardless of characteristics (slowly varying or sharply varying) in a static off‐resonance map

Figures 5 and 6 compare images reconstructed by MaxGIRF reconstruction (L = 8), King's method without static off‐resonance correction, and King's method with static off‐resonance correction for a slice at *z* = 17.5 mm and *z* = 105.0 mm, respectively, from multislice axial spiral spin‐echo imaging of a healthy volunteer at 0.55 T. For nonoblique axial spiral scans, the concomitant fields generate a time‐varying global frequency offset; thus. King's method removed most spatial blurring. King's method with static off‐resonance correction achieved further improvements in regions with slowly varying off‐resonance (Figure 6E), and the sharpness in such regions is comparable with MaxGIRF reconstruction. However, it achieved only minor improvements in regions with sharply varying static off‐resonance (Figure 5E). Because most noniterative off‐resonance methods^61^, ^62^ assume that the static off‐resonance map varies slowly in space, iterative MaxGIRF reconstruction achieved superior performance compared with King's method with static off‐resonance correction, in line with Makhijani and Nayak.^63^ The MaxGIRF reconstruction time was 20 min per slice with a reconstruction matrix size of 320 × 320.

**FIGURE 7:**
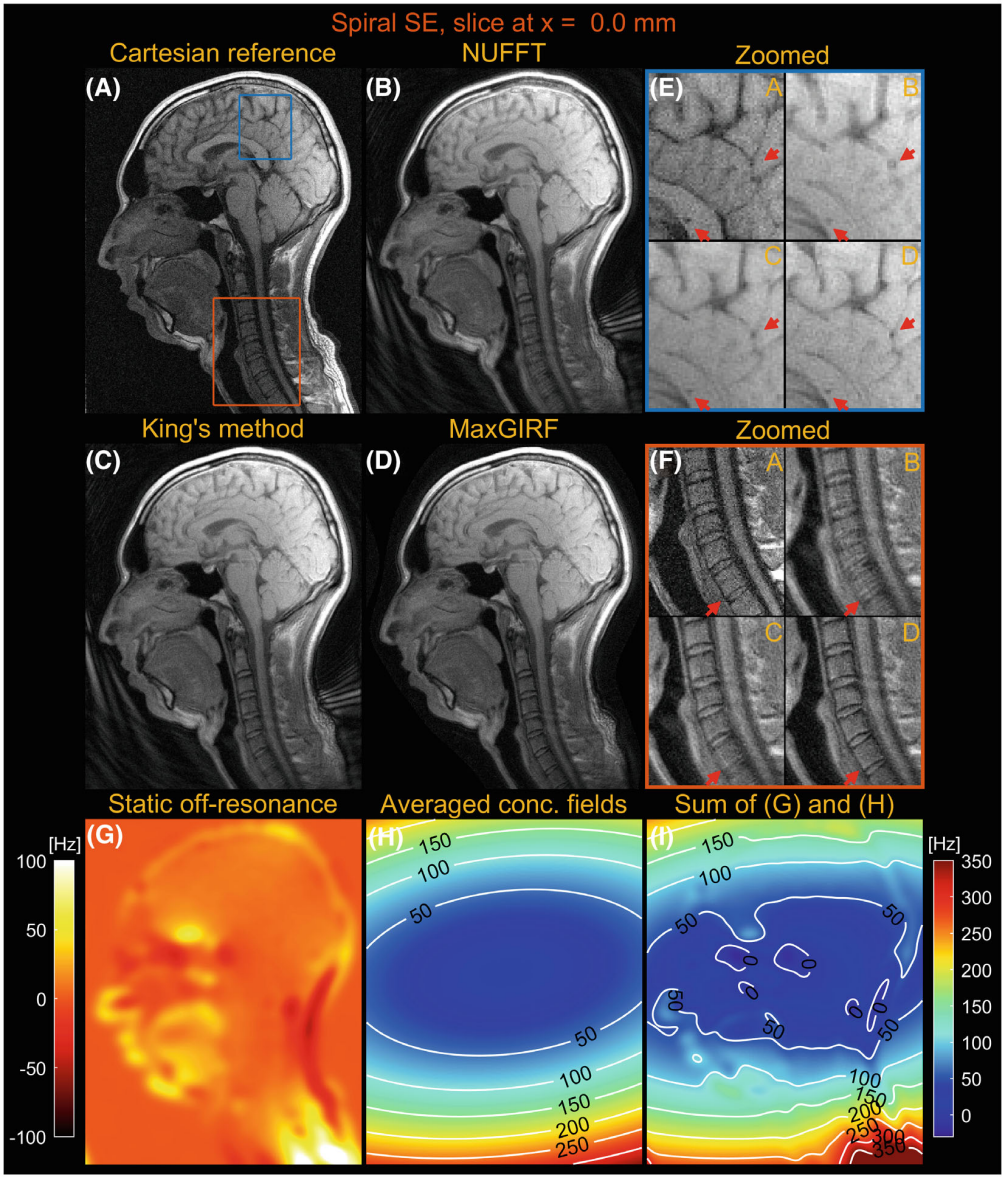
Sagittal spiral spin‐echo imaging of a healthy volunteer at 0.55 T at isocenter (*x* = 0.0 mm). Comparison of image reconstructions using comparator Cartesian spin‐echo image (A), NUFFT reconstruction (B), King's method without static off‐resonance correction (C), and MaxGIRF reconstruction with static off‐resonance correction (Low‐rank approximation L = 30) (D). E, Zoomed‐in image of a brain region (blue box). F, Zoomed‐in image of a neck region (orange box). G, Static off‐resonance map. H, Time‐averaged concomitant fields map. I, Sum of the static off‐resonance map and time‐averaged concomitant fields map. Although MaxGIRF using static off‐resonance is shown in (F), MaxGIRF without static off‐resonance (not shown) is of comparable quality. Thus, this indicates that the improvements in the spine region by MaxGIRF are largely attributed to the methodological difference between King's method and MaxGIRF

**FIGURE 8:**
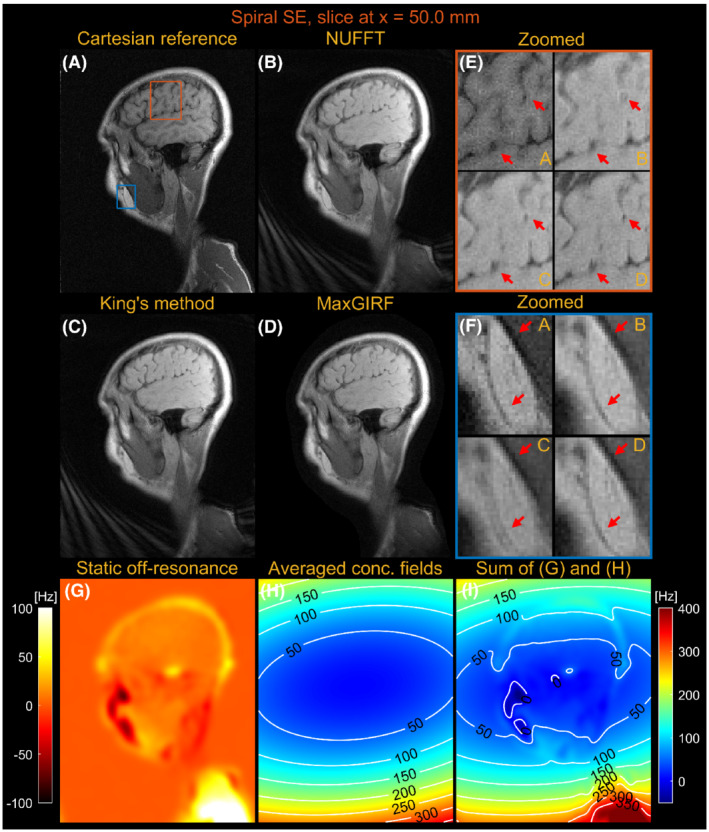
Sagittal spiral spin‐echo imaging of a healthy volunteer at 0.55 T off‐isocenter (*x* = 50.0 mm). Comparison of image reconstructions using comparator Cartesian spin‐echo image (A), NUFFT reconstruction (B), King's method without static off‐resonance correction (C), and MaxGIRF reconstruction with static off‐resonance correction (Low‐rank approximation L = 30) (D). E, Zoomed‐in image of a brain region (orange box). F, Zoomed‐in image (blue box). G, Static off‐resonance map. H, Time‐averaged concomitant fields map. I, Sum of the static off‐resonance map and time‐averaged concomitant fields map. King's method may adversely increase blurring artifacts (eg, blue box) compared with NUFFT reconstruction when the static off‐resonance in a region counteracts the concomitant fields. However, MaxGIRF with static off‐resonance correction correctly handles such regions as shown in (F) and provides “sharper” delineation of brain tissue boundaries in (E) compared with King's method

Figures 7 and 8 compare images reconstructed by NUFFT, King's method (without B_0_ correction), and MaxGIRF (L = 30) for a sagittal slice at *x* = 0.0 mm and *z* = 50.0 mm, respectively, from multislice spiral spin‐echo imaging of a healthy volunteer at 0.55 T. Because the spine region in Figure 7E is reconstructed without static off‐resonance, the improvements by MaxGIRF are solely attributed to the methodological difference between King's method and MaxGIRF. A green box in Figure 8 shows an exemplary region where King's method adversely increases blurring artifacts (compared with NUFFT) when concomitant fields counteract static off‐resonance. In contrast, MaxGIRF with static off‐resonance correction correctly handles such complex situations. The MaxGIRF reconstruction provides “sharper” delineation of brain tissue boundaries in Figure 8E compared with King's method, and its reconstruction time was 3 h per slice with a reconstruction matrix size of 640 × 640.

**FIGURE 9:**
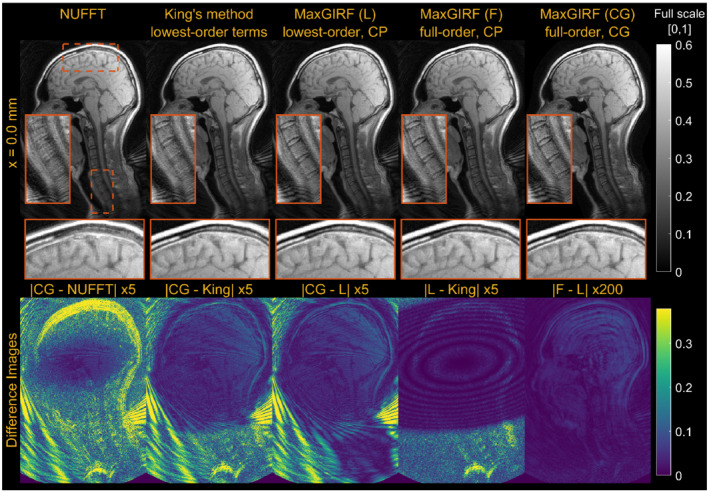
Comparison of reconstruction methods compensating a different number of concomitant field terms. Midsagittal spiral imaging of a healthy volunteer at isocenter at 0.55 T. First column: NUFFT. Second column: King's method without static off‐resonance correction. Third column: Conjugate phase reconstruction–based MaxGIRF using only lowest‐order terms in the concomitant fields. Fourth column: Conjugate phase reconstruction–based MaxGIRF using entire terms in the concomitant fields (full order). Fifth column: Conjugate gradient (CG)–based MaxGIRF using full‐order terms (absolute difference images between reconstructions at the bottom). The GIRF‐predicted gradients were used in all reconstructions. Static off‐resonance correction was not performed, to isolate the difference due to concomitant field correction. The spiral trajectory was designed for 224 × 224 mm^2^ FOV and reconstructed at twice the FOV with the same spatial resolution, which causes the aliasing at the back of the neck

Figure 9 provides a further analysis on MaxGIRF reconstructions including (1) lowest‐order (L) versus full‐order (F) concomitant field compensation; and (2) iterative reconstruction versus noniterative conjugate phase reconstruction (CP). The difference between CP‐based MaxGIRF (F) and CP‐based MaxGIRF (L) was negligible; thus, compensating only lowest‐order terms is sufficient in this case. Given the system's gradient strength and field strength, it is not surprising that the higher‐order terms have a negligible effect. The difference between conjugate gradient–based MaxGIRF (L) and CP‐based MaxGIRF (L) shows primarily aliasing artifacts. The difference between CP‐based MaxGIRF (L) and King's method (King) (both noniterative methods) showed negligible structured artifacts that resemble the shape of concomitant fields at this slice, even in the areas with aliasing artifacts (eg, face). This indicates that both methods perform robustly under the influence of aliasing, and the methodological difference is manifested as the negligible structured artifacts. However, the CP‐based MaxGIRF (L) was only able to compensate strong concomitant fields (> 150 Hz; Figure 7H) near the spine (orange box), whereas King's method showed residual blurring. To further characterize the structured artifacts, noiseless spiral numerical simulations were performed at 0.55 T and 3 T using the same geometry as the human midsagittal scan but with a larger spiral FOV to remove any potential effects of aliasing on the performance of King's method (Supporting Information Figures S1 and S2). The difference image shows that structured artifacts are of identical shape (oval shape centered at isocenter), regardless of field strength (not shown) and distance from isocenter. This simulation indicates that King's method performs well within the boundary of the oval shape but gradually deteriorates beyond this boundary. The size of this oval shape is fixed and not a function of either imaging parameters nor spiral trajectories. The reconstruction times (axial/sagittal) for noniterative MaxGIRF methods and King's method were 40/240 s and 10/20 s, respectively.

## 5 DISCUSSION

We have demonstrated that the MaxGIRF higher‐order encoding matrix approach can be used to simultaneously correct concomitant fields and off‐resonance for spiral acquisitions at 0.55 T. This method uses GIRF‐based gradient waveform corrections to accurately calculate spatiotemporally varying concomitant field estimates and static off‐resonance maps to generate a hybrid signal equation with variables in the physical and logical coordinate system for image reconstruction. We characterized the accuracy of a low‐rank approximation of higher‐order encoding matrices to improve reconstruction times with NRMSEs, and implement a randomized SVD to mitigate memory requirements. The MaxGIRF approach provides improved sharpness in regions with large concomitant fields (including off‐isocenter) and/or nonzero static off‐resonance, compared with King's method. The effectiveness of the proposed method has been demonstrated with numerical simulations, phantom, and in vivo human spiral acquisitions.

Here, we applied MaxGIRF to spiral imaging using a lower field strength (0.55 T) MRI system. Concomitant field effects are increased at lower field strength, higher gradient amplitudes, longer readouts, and distance from isocenter. Therefore, this method is generalizable for several other MRI applications including systems with gradient inserts, permitting higher peak gradient amplitude, large FOV imaging, and all field strengths.

The phantom‐based GIRF measurements used by the MaxGIRF approach can be a viable alternative to NMR field probes when gradient nonlinearity is not too severe, gradient systems are approximately linear time‐invariant over the duration of a scan, and models of concomitant fields are well‐matched to real measurements. We presumed zero gradient nonlinearity but noticed image distortions both in Cartesian and spiral reconstructions (eg, sagittal slice of the NIST phantom). The concomitant fields derived without gradient nonlinearity may be sufficient for FOVs used in the current study, but a further investigation is required for large FOV spiral acquisitions (eg, cardiac, abdominal, or fetal imaging), especially in large‐bore MR systems or MR systems with a high‐performance gradient insert.^33^, ^64^, ^65^, ^66^ Because gradient nonlinearity along each gradient direction can be modeled by a product of spherical harmonics^67^, ^68^ and a linear gradient normalized by a reference gradient,^69^, ^70^ concomitant field terms incorporating the spherical harmonics expansion (possibly up to ninth order)^71^ of gradient nonlinearity could be derived following the approach described in Testud et al.^17^ Because gradient nonlinearity and a new set of concomitant fields under gradient nonlinearity are a function of linear gradients, both could be predicted by phantom‐based GIRFs with high accuracy and incorporated within the MaxGIRF framework.

An optimal rank criterion should depend on the specific MR application. In this study, we choose an optimal rank that gives less than 2% error in both magnitude and phase NRMSEs. This stringent requirement can be relaxed when signal intensity in regions with high off‐resonance (eg, fat regions) may not be of interest. In the case of water–fat‐separated imaging or when fat suppression is used, the static off‐resonance map may become smoother because the discrete water/fat chemical shift is removed. In this case, the optimal rank may be lower because singular values of smoother images decay more rapidly.^72^

One notable advantage of the MaxGIRF approach is that it can be easily adapted to many clinical sites without NMR field probes. The MaxGIRF approach only requires good analytic models of concomitant fields and GIRFs measured with a simple pulse sequence and a spherical phantom. Because analytic expressions of concomitant fields for asymmetric gradient coils can be derived,^32^ the MaxGIRF approach would be applicable to clinical systems with asymmetric gradient coils that have well‐documented analytic expressions. Pulse sequences for GIRF measurements can be developed and shared via a vendor‐independent pulse sequence framework (eg, Pulseq and TOPPE).^73^, ^74^ This would enable clinical sites without expertise in sequence programming to obtain GIRF measurements on their own scanners. Note that a pulse sequence for GIRF measurements described in Vannesjo et al^22^ is provided by TOPPE.^75^ Because of its simple reconstruction procedure, the MaxGIRF approach can be easily integrated into any existing gridding or NUFFT based non‐Cartesian reconstruction routines provided in open‐source reconstruction platforms such as BART,^76^ Gadgetron,^77^ and GPI.^78^ Therefore, reconstruction software can potentially be shared among sites without difficulty.

This work has several limitations. We did not consider acquisitions in which an accumulated concomitant phase affects the net phase of spin isochromats after following excitation or refocusing pulses. This specifically includes balanced SSFP and fast spin‐echo sequences, each of which may require additional assumptions (eg, a perfect 180 refocusing pulse for fast spin echo) or additional pulse‐sequence modifications to formulate a tractable forward model that can be solved with an extension of the MaxGIRF framework.

Another drawback is reconstruction time. The SVD needs to be computed for each subject with a unique static off‐resonance map and whenever a slice prescription is changed. The SVD computation time was 1 min and 6 min for axial and sagittal scans, respectively, using a non‐parallelized implementation. However, this long computation time could be reduced by switching from a CPU‐based randomized SVD implementation (used in this study) to one implemented in parallel architectures such as GPUs. The other computation bottleneck is L repetitions of NUFFTs. Because the current *MATLAB* implementation does not use parallel computing via multicore CPUs, MaxGIRF reconstruction is relatively slow (L times longer than conjugate gradient–based SENSE). This limitation could be partially overcome with simultaneous computations of $L\times N_{i}$ NUFFTs using multiple GPUs. This may be particularly beneficial for 3D spiral and/or very high resolution spiral scans.

## 6 CONCLUSIONS

We demonstrate a higher‐order image reconstruction method, called MaxGIRF, that incorporates concomitant fields and GIRF‐based gradient waveform prediction for spoiled gradient‐echo and spin‐echo spiral imaging. Simulations indicate that MaxGIRF successfully mitigates local blurring caused by concomitant fields at various field strengths and distances from isocenter. The MaxGIRF reconstruction was able to mitigate concomitant fields both in phantom and in vivo brain spiral imaging at 0.55 T, superior to the most notable existing solution. Including an accurate static off‐resonance map further improves its performance in regions with large static off‐resonance. The impact of this method is greatest when imaging with longer readouts, high gradient amplitudes, and/or at lower field strength.

## Supporting information


**Text S1.** Coordinate transformations.
**Text S2.** Randomized SVD.
**Figure S1**. Comparison between MaxGIRF andKing's method for sagittal orientation using noiseless numerical simulations.(1st column) Conjugate phase reconstruction‐based MaxGIRF using onlylowest‐order terms in the concomitant fields. (2nd column) King'smethod without static off‐resonance correction. (3rd column) Theabsolute difference between MaxGIRF (L) and King's method. (4th column) Time averaged concomitant fields map. Noiseless numerical simulationswere performed using a slice (1st row) at isocenter and (2nd row) 100‐mm distance from isocenter. The FOV of spiral waveforms was set to 30cm and the image was reconstructed at twice the FOV. The reconstruction matrixwas 512 x 512 and the matrix size of a displayed image was 320 x 320, givingrise to 30 cm * 2 / 512 * 320 = 37.5 cm displayed FOV.
**Figure S2**. Comparison between MaxGIRF and King's method for coronal orientation using noiseless numerical simulations.The simulations were identical to those in Supporting Information Figure S1 except that the slice offsetdirection was the y‐axis instead of x‐axis. See Supporting Information Figure S1 for details.

## Data Availability

The code and sample data (ISMRMRD format) that support the findings of this study are openly available in GitHub at https://www.github.com/usc‐mrel/lowfield_maxgirf and https://www.github.com/usc‐mrel/nlinv_estimation.

## APPENDIX A

The nonlinear signal model $G\left(\hat{x}\right)$ is first linearized with the Taylor expansion around the current estimate ${\hat{x}}_{n}$ as follows:

(A1)
$$G\left({\hat{x}}_{n}+d\hat{x}\right)\approx G\left({\hat{x}}_{n}\right)+\hat{D}G\left({\hat{x}}_{n}\right)d\hat{x},$$

where $\hat{D}G\left({\hat{x}}_{n}\right)=\left[\frac{\partial G}{\partial \hat{\rho }}\;\frac{\partial G}{\partial \hat{\mathrm{\Delta f}}}\right]$ is the Fréchet derivative of G evaluated at ${\hat{x}}_{n}$. Substituting $\hat{x}$ with ${\hat{x}}_{n}+d\hat{x}$ in Eq. 18 leads to the cost function that provides the update $d\hat{x}$ for the nth Gauss‐Newton iteration ${\hat{x}}_{n+1}={\hat{x}}_{n}+d\hat{x}$ as follows:

(A2)
$$\begin{array}{cc} \Phi \left(d\hat{x}\right) & =\underset{d\hat{x}}{\text{argmin}}{\left\|y-G\left({\hat{x}}_{n}\right)-\hat{D}G\left({\hat{x}}_{n}\right)d\hat{x}\right\|}_{\ell_{2}}^{2}\\ & \;+\alpha_{n}{\left\|{\hat{x}}_{n}+d\hat{x}-{\hat{x}}_{0}\right\|}_{\ell_{2}}^{2}. \end{array}$$

Equation A2 can be simplified to

(A3)
$$\begin{array}{cc} & \left({\left[\hat{D}G\left({\hat{x}}_{n}\right)\right]}^{H}\hat{D}G\left({\hat{x}}_{n}\right)+\alpha_{n}I_{2N}\right)d\hat{x}\\ & \;={\left[\hat{D}G\left({\hat{x}}_{n}\right)\right]}^{H}\left(y-G\left({\hat{x}}_{n}\right)\right)+\alpha_{n}\left({\hat{x}}_{n}-{\hat{x}}_{0}\right). \end{array}$$

Because $\hat{D}G\left(\hat{x}\right)≜\frac{d}{d\hat{x}}G\left(\hat{x}\right)=\frac{d}{d\hat{x}}F\left(x\right)=\frac{d}{dx}F\left(x\right)\frac{dx}{d\hat{x}}=DF\left(x\right)W$, Eq. A3 can be expressed in terms of $DF\left(x_{n}\right)$ and solved with LSQR as follows:

(A4)
$$\begin{array}{cc} & \left(W^{H}{\left[DF\left(x_{n}\right)\right]}^{H}DF\left(x_{n}\right)W+\alpha_{n}I_{2N}\right)d\hat{x}\\ & \;=W^{H}{\left[DF\left(x_{n}\right)\right]}^{H}\left(y-F\left(x_{n}\right)\right)+\alpha_{n}\left({\hat{x}}_{n}-{\hat{x}}_{0}\right). \end{array}$$

The regularization parameter is set to decrease per iteration as $\alpha_{n}=\alpha_{0}q^{n}$, where q=2/3, until it reaches a minimum regularization parameter $\alpha_{\min}$. The data vector $y\in {\mathbb{C}}^{N_{e}N\times 1}$ is scaled to have “100.0 L2 norm,”^79^ and scaling of unknowns is not used. The derivative operator $DF\left(x\right)\in {\mathbb{C}}^{N_{e}N\times 2N}$ is defined as

(A5)
$$\begin{array}{cc} & DF\left(x\right)=\left[\begin{array}{cc} \frac{\partial F}{\partial \rho } & \frac{\partial F}{\partial \mathrm{\Delta f}} \end{array}\right]=\left[\begin{array}{cc} \frac{\partial F_{1}}{\partial \rho } & \frac{\partial F_{1}}{\partial \mathrm{\Delta f}}\\ \vdots & \vdots \\ \frac{\partial F_{N_{e}}}{\partial \rho } & \frac{\partial F_{N_{e}}}{\partial \mathrm{\Delta f}} \end{array}\right]\\ & =\left[\begin{array}{cc} \mathrm{diag}\left(\exp\left(j2\pi \mathrm{\Delta f}{\mathrm{TE}}_{1}\right)\right) & \mathrm{diag}\left(\rho \right)\mathrm{diag}\left(\exp\left(j2\pi \mathrm{\Delta f}{\mathrm{TE}}_{1}\right)\right)\left(j2\pi {\mathrm{TE}}_{1}\right)\\ \vdots & \vdots \\ \mathrm{diag}\left(\exp\left(j2\pi \mathrm{\Delta f}{\mathrm{TE}}_{N_{e}}\right)\right) & \mathrm{diag}\left(\rho \right)\mathrm{diag}\left(\exp\left(j2\pi \mathrm{\Delta f}{\mathrm{TE}}_{N_{e}}\right)\right)\left(j2\pi {\mathrm{TE}}_{N_{e}}\right) \end{array}\right]. \end{array}$$

Using Eq. A5, the matrix–vector product $\mathrm{dy}=DF\left(x\right)\mathrm{Wd}\hat{x}\in {\mathbb{C}}^{N_{e}N\times 1}$ is calculated as

(A6)
$$\begin{array}{cc} \mathrm{dy} & =\left[\begin{array}{c} dy_{1}\\ \vdots \\ dy_{N_{e}} \end{array}\right]=DF\left(x\right)\left[\begin{array}{c} \mathrm{d\rho}\\ \mathrm{d\Delta f} \end{array}\right]\\ & =\left[\begin{array}{c} \mathrm{diag}\left(\psi_{1}\right)\mathrm{d\rho}+\mathrm{diag}\left(\rho \right)\mathrm{diag}\left(\psi_{1}\right)\left(j2\pi {\mathrm{TE}}_{1}\right)\mathrm{d\Delta f}\\ \vdots \\ \mathrm{diag}\left(\psi_{N_{e}}\right)\mathrm{d\rho}+\mathrm{diag}\left(\rho \right)\mathrm{diag}\left(\psi_{N_{e}}\right)\left(j2\pi {\mathrm{TE}}_{N_{e}}\right)\mathrm{d\Delta f} \end{array}\right], \end{array}$$

where we define $\psi_{m}=\exp\left(j2\pi \mathrm{\Delta f}{\mathrm{TE}}_{m}\right)$. Similarly, the matrix–vector product $\mathrm{dx}={\left[DF\left(x\right)\right]}^{H}\mathrm{dy}\in {\mathbb{C}}^{2N\times 1}$ involving the adjoint of the derivative operator can be calculated as

(A7)
$$\begin{array}{cc} \mathrm{dy} & =\left[\begin{array}{c} \mathrm{d\rho}\\ \mathrm{d\Delta f} \end{array}\right]={\left[DF\left(x\right)\right]}^{H}\left[\begin{array}{c} dy_{1}\\ \vdots \\ dy_{N_{e}} \end{array}\right]\\ & =\left[\begin{array}{c} \sum_{m=1}^{N_{e}}\mathrm{diag}\left(\psi_{m}^{*}\right)dy_{m}\\ R\left\{\sum_{m=1}^{N_{e}}\mathrm{diag}\left(\rho \right)\mathrm{diag}\left(\psi_{m}^{*}\right)\left(-j2\pi {\mathrm{TE}}_{m}\right)dy_{m}\right\} \end{array}\right], \end{array}$$

where R{⋅} denotes the real operator that keeps only the real part of a complex‐valued input.
