# Supplementary material for: MaxGIRF: Image reconstruction incorporating concomitant field and gradient impulse response function effects
Source: Magn Reson Med. 2022 Apr 21;88(2):691–710. doi: 10.1002/mrm.29232 (PMC9232904; doi:10.1002/mrm.29232)
Supplement: Supplementary file 1 — Text S1. Coordinate transformations. Text S2. Randomized SVD. Figure S1. Comparison between MaxGIRF andKing's method for sagittal orientation using noiseless numerical simulations.(1st column) Conjugate phase reconstruction‐based MaxGIRF using onlylowest‐order terms in the concomitant fields. (2nd column) King'smethod without static off‐resonance correction. (3rd column) Theabsolute difference between MaxGIRF (L) and King's method. (4th column) Time averaged concomitant fields map. Noiseless numerical simulationswere performed using a slice (1st row) at isocenter and (2nd row) 100‐mm distance from isocenter. The FOV of spiral waveforms was set to 30cm and the image was reconstructed at twice the FOV. The reconstruction matrixwas 512 x 512 and the matrix size of a displayed image was 320 x 320, givingrise to 30 cm * 2 / 512 * 320 = 37.5 cm displayed FOV. Figure S2. Comparison between MaxGIRF and King's method for coronal orientation using noiseless numerical simulations.The simulations were identical to those in Supporting Information Figure S1 except that the slice offsetdirection was the y‐axis instead of x‐axis. See Supporting Information Figure S1 for details. [file MRM-88-691-s001.docx]

**SUPPORTING INFORMATION TEXT S1**

**Coordinate transformations**

When Cartesian $\mathbf{k}$-space data in ISMRMRD format (1) is read in a programming platform (MATLAB) with the ISMRMRD reader, the readout (**RO**) direction is placed along the first dimension (row), phase encoding (**PE**) and slice selection (**SL**) directions along the second (column) and third (slice) dimensions, respectively. The readout direction can be determined from Cartesian $\mathbf{k}$-space data with intended oversampling along the readout direction. Note that the zero frequency of a $\mathbf{k}$-space matrix is placed at the FFT/NUFFT center of an $N_{1}\times N_{2}\times N_{3}$ matrix, i.e., $\left( \left\lfloor\frac{N_{1}}{2} \right\rfloor+1,\left\lfloor\frac{N_{2}}{2} \right\rfloor+1,\left\lfloor\frac{N_{3}}{2} \right\rfloor+1 \right),$ for index starting from 1. Note that this center should be the common origin among $\mathbf{k}$-space trajectories in different coordinate systems. Let us define a new coordinate system called matrix coordinate system, where each voxel with matrix index $\left( I_{1},I_{2},I_{3} \right)$ is assigned spatial coordinates $\mathbf{r}_{R}$ (in m)

|  | $\mathbf{r}_{R}=\left[ \begin{matrix} r \\ c \\ s \end{matrix} \right]=\left[ \begin{matrix} \left( I_{1}-\left( \left\lfloor\frac{N_{1}}{2} \right\rfloor+1 \right) \right){\Delta V}_{1} \\ \left( I_{2}-\left( \left\lfloor\frac{N_{2}}{2} \right\rfloor+1 \right) \right){\Delta V}_{2} \\ \left( I_{3}-\left( \left\lfloor\frac{N_{3}}{2} \right\rfloor+1 \right) \right){\Delta V}_{3} \end{matrix} \right],$ | [S1] |
| --- | --- | --- |

where each index $I_{\mathcal{l}}$ ranges from $1 \mathrm{to}N_{\mathcal{l}}$ and ${\Delta V}_{\mathcal{l}}$ is the voxel size for $\mathcal{l=}1,2,3$. The spatial coordinates of a voxel in the matrix coordinate system (i.e., matrix coordinates) is related to the spatial coordinates in the logical coordinate system (i.e., logical coordinates) $\mathbf{r}_{L}=\left[ PE,RO,SL \right]^{T}$ via a transformation matrix $\mathbf{R}_{\mathrm{RtoL}}$ as follows:

|  | $\mathbf{r}_{L}=\underset{\mathbf{R}_{\mathrm{RtoL}}}{\underbrace{\left[ \begin{matrix} 0 & 1 & 0 \\ 1 & 0 & 0 \\ 0 & 0 & 1 \end{matrix} \right]}}\mathbf{r}_{R}.$ | [S2] |
| --- | --- | --- |

However, depending on a vendor, the polarity of gradients (i.e., gradient sign flip) can be implicitly changed depending on the scan orientation and the rotation angle of a scan plane and thus the RO and PE directions could be flipped. This requires subsequent $\mathbf{k}$-space data flipping and additional sign changes in the columns of $\mathbf{R}_{\mathrm{LtoP}}$ may be required. Note that calculating $\mathbf{R}_{\mathrm{LtoP}}$ and $\mathbf{r}_{P,offset}$ may involve other intermediate coordinate systems (e.g., patient coordinate system) and the details of coordinate transformations are proprietary information. Reverse engineering the sign flip requires substantial efforts and as a validation step, user-defined patient coordinates can be compared with the patient coordinates provided by vendor DICOM. It is very important to use the same FT/IFT convention as the vendor of your MR system when performing MaxGIRF reconstruction and reverse engineering coordinate transformations with DICOM information. When performing NUFFT, $\mathbf{k}$-space trajectories are transformed from the logical coordinate system to the matrix coordinate system. Using Equation S2, $\mathbf{k}$-space trajectories in the matrix coordinate system are obtained by $\mathbf{k}_{R, i}\left( t \right)={\mathbf{(R}_{\mathrm{RtoL}}\boldsymbol{)}}^{\boldsymbol{-1}}\boldsymbol{\cdot}\mathbf{k}_{L, i}\left( t \right)$.

**SUPPORTING INFORMATION TEXT S2**

**Randomized SVD**

A conventional SVD algorithm operates on an entire matrix. This approach may be slow and even infeasible when storing higher-order encoding matrices requires significant amount of memory. Our approach employs a memory-efficient implementation (similar to (1)) of a randomized SVD (2) to handle such high memory demand when longer readouts and high spatial resolution are used. The randomized SVD was especially developed to handle large matrices with a modern scalable architecture. Many randomized SVD algorithms are based on a key idea of randomized numerical linear algebra (3), briefly summarized as follows: when a data matrix $\mathbf{H}\in\mathbb{C}^{N_{k}\times N}$ is multiplied by a random projection matrix $\mathbf{P}\in\mathbb{C}^{N\times r}$, the smaller sketch matrix $\mathbf{Z=H}\mathbf{P}\in\mathbb{C}^{N_{k}\times r}$ preserves rank properties of the original data matrix with high probability. Following this principle, a randomized SVD is calculated with the following procedure (4) : 1) a target rank $r$ slightly larger than $L$ is defined (known as oversampling, e.g., $r=L+5)$; 2) the column space of a higher-order encoding matrix is sampled with a Gaussian random projection matrix: $\mathbf{Z}_{i}\mathbf{=}\mathbf{H}_{i}\mathbf{P}_{i}$; 3) the QR decomposition of $\mathbf{Z}_{i}$ is performed to compute the column space of $\mathbf{Z}_{i}$: $\mathbf{Z}_{i}\mathbf{=} \mathbf{Q}_{i}\mathbf{R}_{i}$; 4) a higher-order encoding matrix is projected onto $\mathbf{Q}_{i}$**:** ${\mathbf{Y}_{i}\mathbf{=Q}}_{\boldsymbol{i}}^{T}\mathbf{H}_{i}$, 5) compute the SVD of $\mathbf{Y}_{i}$: $\mathbf{Y}_{i}=\mathbf{U}_{\boldsymbol{Y}_{i}}\boldsymbol{\Sigma}_{i}{\mathbf{V}_{i}}^{H}$; 6) project back to the original dimension: ${{\mathbf{H}_{i}\boldsymbol{\approx Q}}_{i}\mathbf{Y}}_{i}={\mathbf{Q}_{i}\mathbf{U}}_{\boldsymbol{Y}_{i}}\boldsymbol{\Sigma}_{i}{\mathbf{V}_{i}}^{H}\boldsymbol{=}\mathbf{U}_{i}\boldsymbol{\Sigma}_{i}{\mathbf{V}_{i}}^{H}$. Because storing an entire higher-order encoding matrix in memory may not be feasible, a higher-order encoding matrix and a random projection matrix are first divided into submatrices:

|  | $\mathbf{H}_{i}=\left[ \begin{matrix} \mathbf{H}_{i}^{1} & \boldsymbol{\cdots} & \mathbf{H}_{i}^{s} \end{matrix} \right] \mathrm{and} \mathbf{P}_{i}=\left[ \begin{matrix} \mathbf{P}_{i}^{1} \\ \vdots\\ \mathbf{P}_{i}^{s} \end{matrix} \right].$ | [S3] |
| --- | --- | --- |

Only a pair of submatrices $\left( \mathbf{H}_{i}^{j},\mathbf{P}_{i}^{j} \right)$ and its product are computed in memory and this procedure continues until the last pair: $\mathbf{Z}_{i}\mathbf{=}\sum_{j\boldsymbol{=1}}^{s} \mathbf{H}_{i}^{j}\mathbf{P}_{i}^{j}$.

**REFERENCES**

1. Yang M, Ma D, Jiang Y, et al. Low Rank Approximation Methods for MR Fingerprinting With Large Scale Dictionaries. 2018;2400:2392–2400 doi: 10.1002/mrm.26867.

2. Halko N, Martinsson PG, Tropp JA. Finding structure with randomness: Probabilistic algorithms for constructing approximate matrix decompositions. SIAM Rev. 2011;53:217–288 doi: 10.1137/090771806.

3. Drineas P, Mahoney MW. Randomization offers new benefits for large-scale linear algebra computations. Commun. Acm 2016;59.

4. Erichson NB, Voronin S, Brunton SL, Kutz JN. Randomized matrix decompositions using R. J. Stat. Softw. 2019;89 doi: 10.18637/jss.v089.i11.


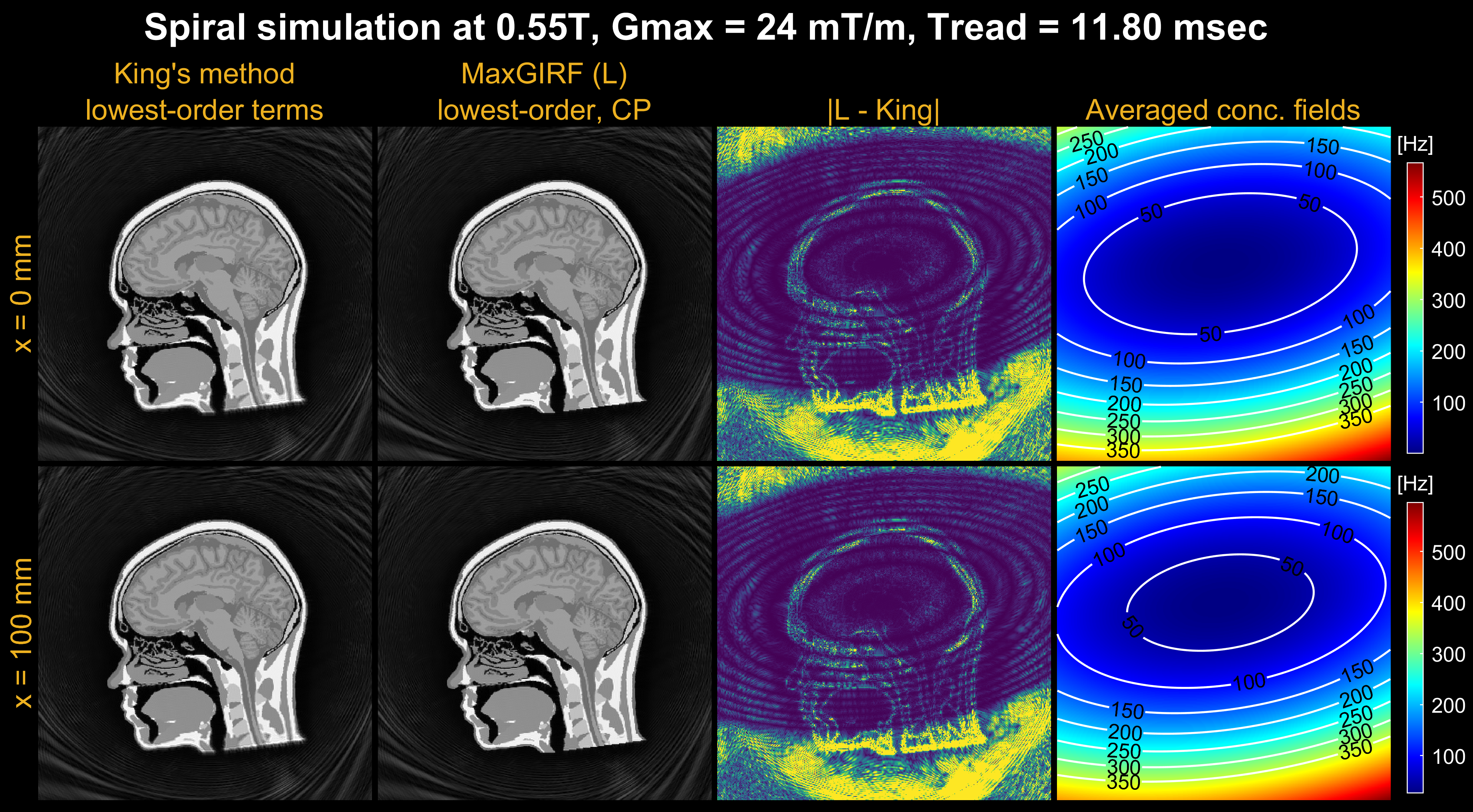


**Supporting Information Figure S1.** Comparison between MaxGIRF and King’s method for sagittal orientation using noiseless numerical simulations. (1^st^ column) Conjugate phase reconstruction-based MaxGIRF using only lowest-order terms in the concomitant fields. (2^nd^ column) King’s method without static off-resonance correction. (3^rd^ column) The absolute difference between MaxGIRF (L) and King’s method. (4^th^ column) Time averaged concomitant fields map. Noiseless numerical simulations were performed using a slice (1^st^ row) at isocenter and (2^nd^ row) 100-mm distance from isocenter. The FOV of spiral waveforms was set to 30 cm and the image was reconstructed at twice the FOV. The reconstruction matrix was 512 x 512 and the matrix size of a displayed image was 320 x 320, giving rise to 30 cm * 2 / 512 * 320 = 37.5 cm displayed FOV.


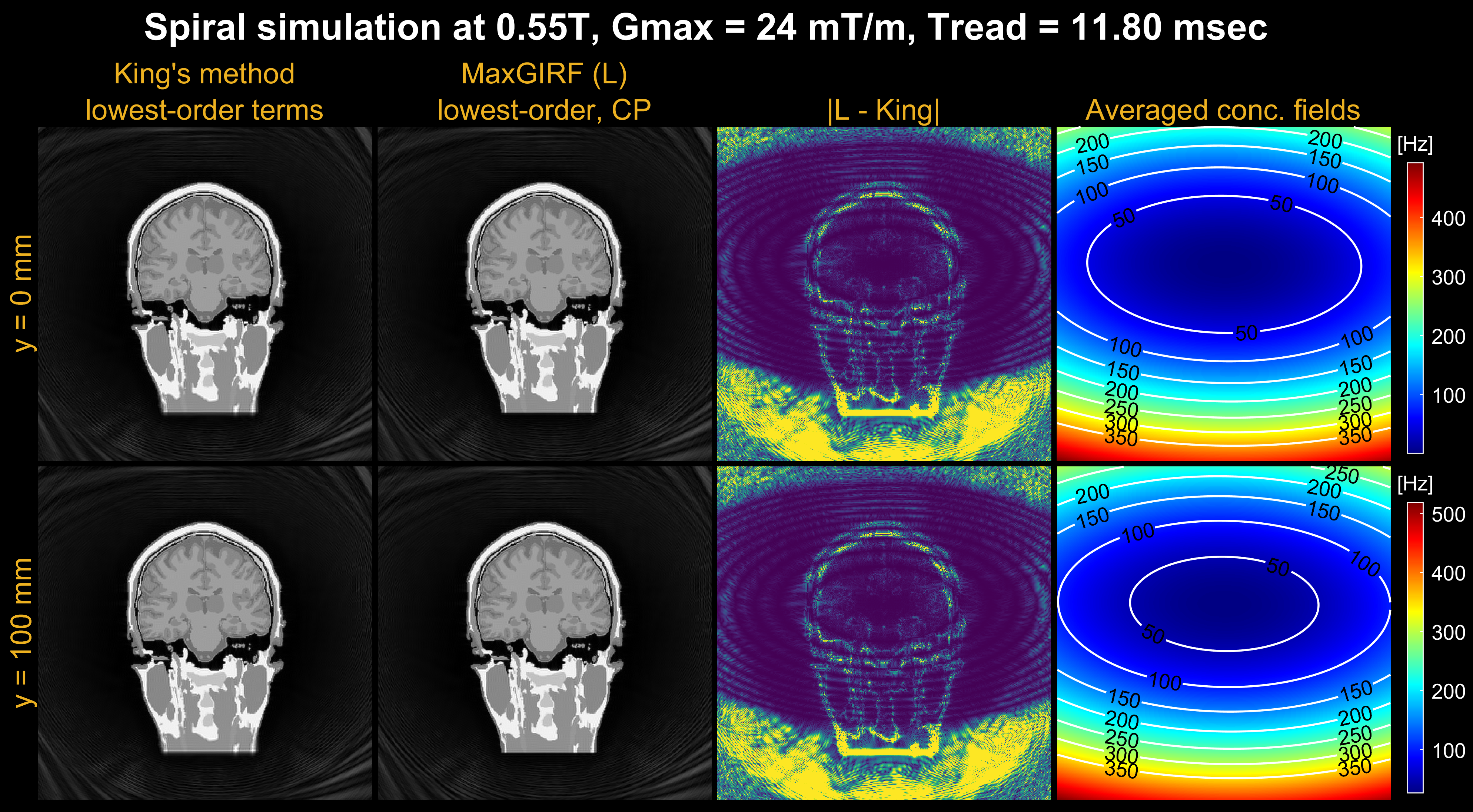


**Supporting Information Figure S2.** Comparison between MaxGIRF and King’s method for coronal orientation using noiseless numerical simulations. The simulations were identical to those in **Supporting Information Figure S1** except that the slice offset direction was the y-axis instead of x-axis. See **Supporting Information Figure S1** for details.
